# Supplementary material for: Whole-cell modeling in yeast predicts compartment-specific proteome constraints that drive metabolic strategies
Source: Nat Commun. 2022 Feb 10;13:801. doi: 10.1038/s41467-022-28467-6 (PMC8831649; doi:10.1038/s41467-022-28467-6)
Supplement: Supplementary file 1 — Supplementary Information [file 41467_2022_28467_MOESM1_ESM.pdf]

Supplementary Material for

Whole-cell modeling in yeast predicts compartment-specific proteome constraints that drive metabolic strategies

Ibrahim E. Elsemman, Angelica Rodriguez Prado, Pranas Grigaitis *et al.*

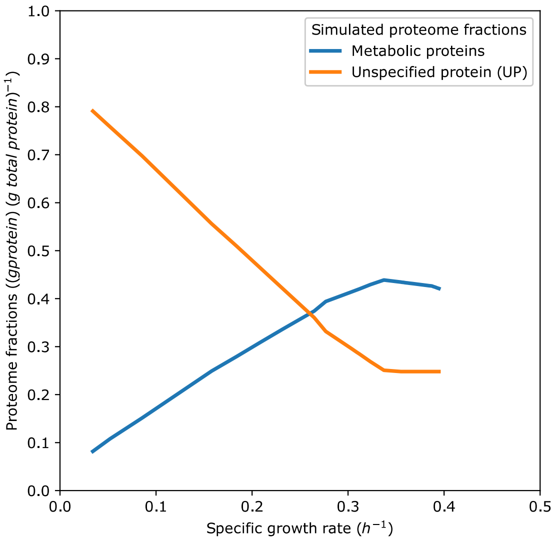

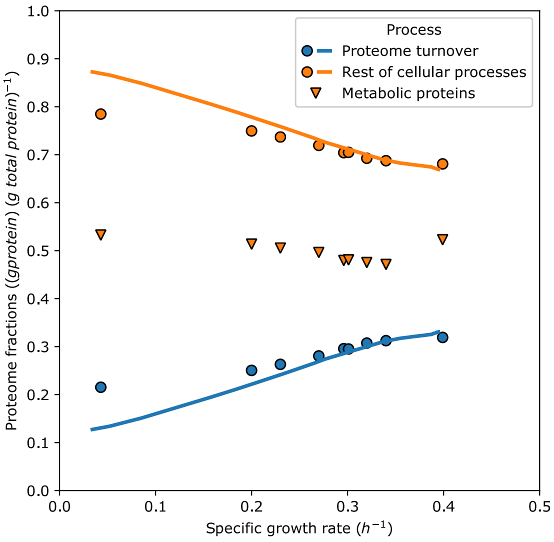

Supplementary Figure 1. Computed protein fractions as a function of growth rate. Left, predicted fractions of the unspecified protein (orange) and metabolic proteins (blue); right, fractions of proteome turnover-associated proteins (blue) and proteins, participating in other cellular processes (orange).

The dots represent experimentally determined proteome fractions from glucose-limited chemostats (at $D = 0.20$ to $0.34\, h^{-1}$) and trehalose- and glucose-excess batch cultivations ($\mu = 0.043$ and $\mu = 0.399\, h^{-1}$; left-most and right-most points, respectively), and lines are model predictions in glucose-limited conditions. The annotations for the "Proteome turnover" class were collected by the Authors (Table S1); for "Metabolic proteins", we used KEGG ontology annotations for processes related to metabolism.

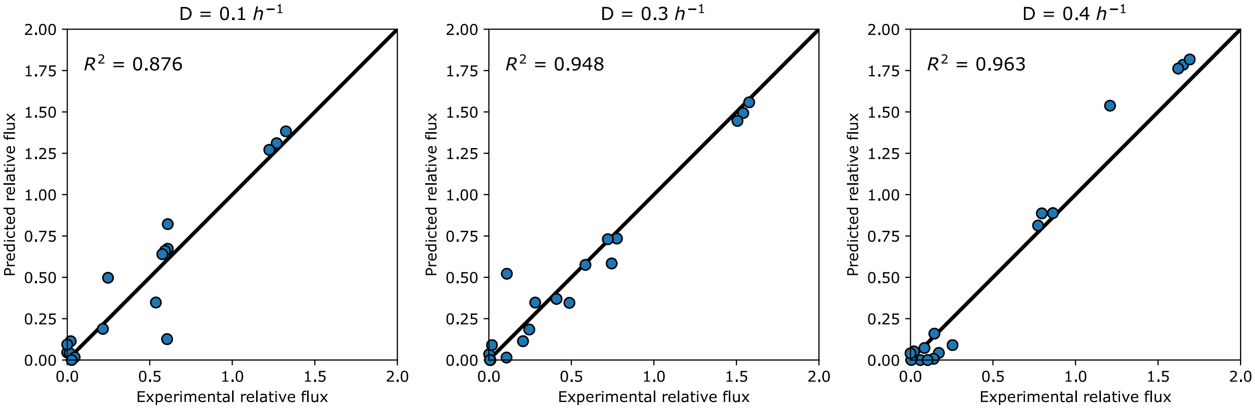

Supplementary Figure 2. Comparison of experimentally determined (abscissa) and predicted (ordinate) fluxes in central carbon metabolism of glucose-limited *S. cerevisiae*. Simulations of glucose-limited growth were performed by varying the hexose transporter saturation, as for the Figure 2d of the main text. Experimental data for the intracellular fluxes at three dilution rates ($D = 0.1$, 0.3 and $0.4\ h^{-1}$) were taken from[1]. Flux values were normalized to those of the hexokinase reaction.

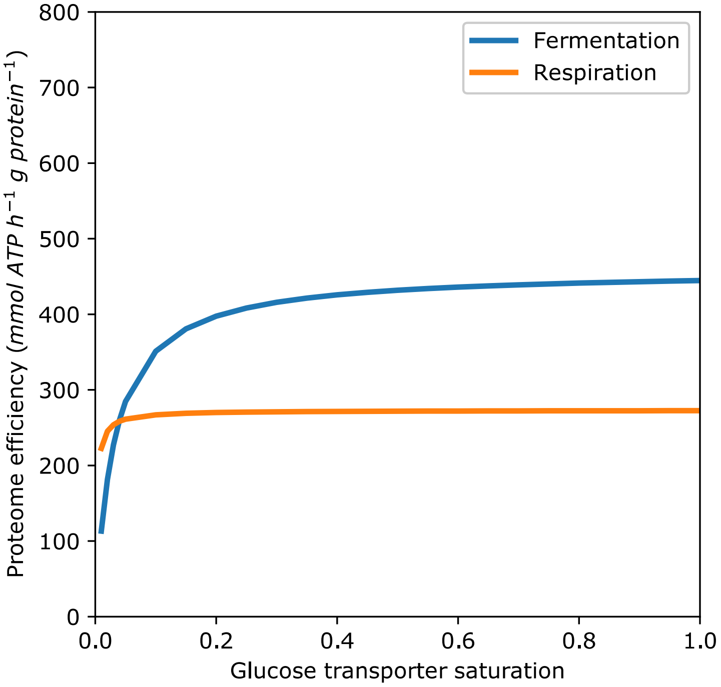

Supplementary Figure 3. Dependence of the proteome efficiency (measured in $mmol\ ATP/g\ protein/h$) for energy harvesting from glucose through respiration and fermentation pathways on the saturation of the glucose transporter. In brief, we compiled information on the molecular weights of the proteins that participate in glycolysis, TCA cycle and oxidative phosphorylation, together with respective turnover values. Then we computed the amounts of each protein (in grams) needed in order to sustain a flux of $1\ mmol\ ATP/h$ using either respiratory of fermentative pathway under different saturation of glucose transporter, as described previously[2]. The final value of the proteome efficiency was computed by evaluating the flux of ATP production per gram of protein invested.

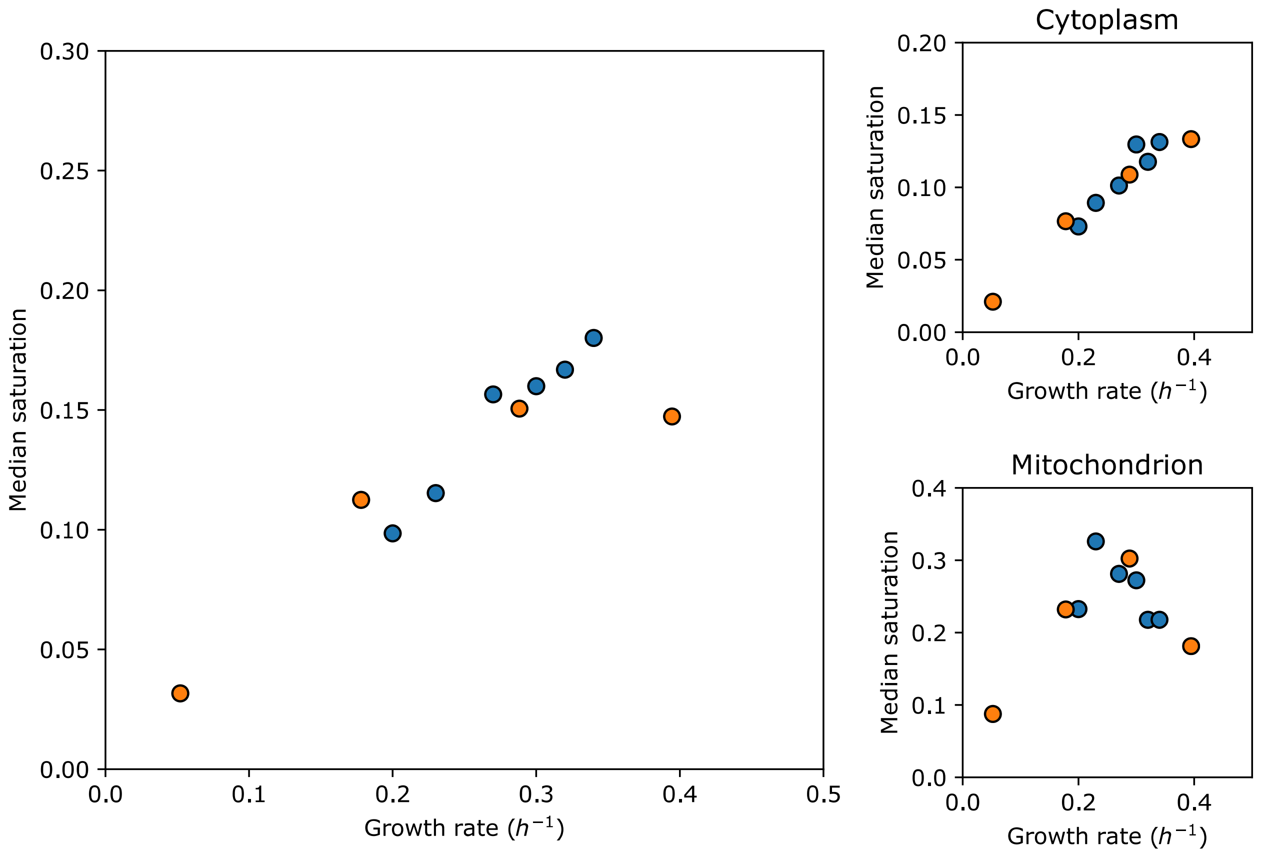

Supplementary Figure 4. Growth-rate dependence of the median saturation factor of metabolic enzymes. Blue points, glucose-limited chemostat cultures; orange points, sugar excess cultures. The saturation factor was determined as a ratio between the expression level predicted by the pcYeast model vs. the experimentally determined expression level from quantitative proteomics measurements. Ratios were taken into consideration only if the protein abundance was both present and nonzero in both datasets. All proteins with a saturation factor higher than 1 were assigned a factor of 1.1.

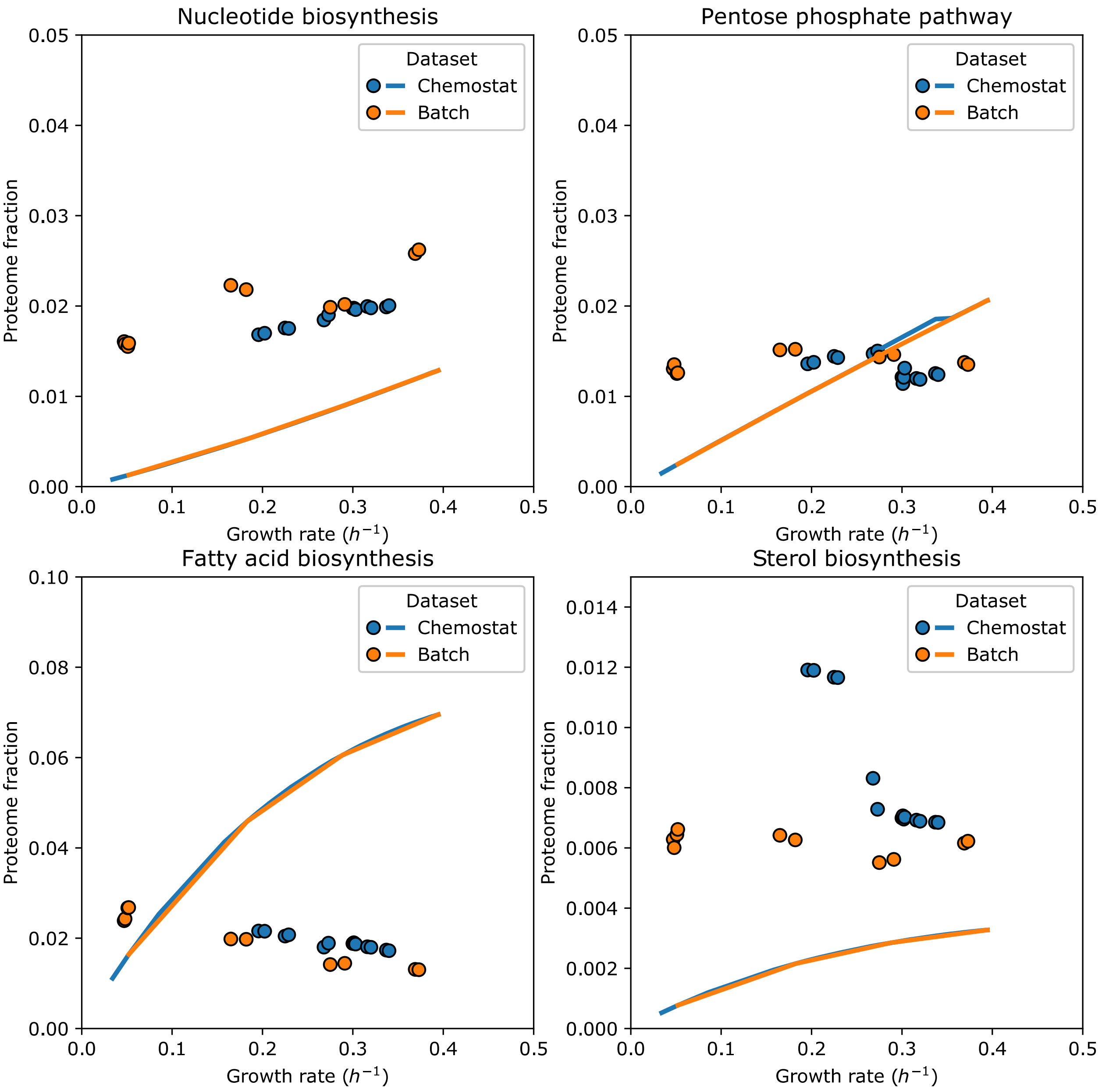

Supplementary Figure 5. Comparison of experimentally determined (circles) and predicted (lines) proteome fractions occupied by proteins in some biosynthetic pathways). Pathway-protein mappings (Table S1) were manually curated, using the reference proteome of *S. cerevisiae* from the UniProt database.

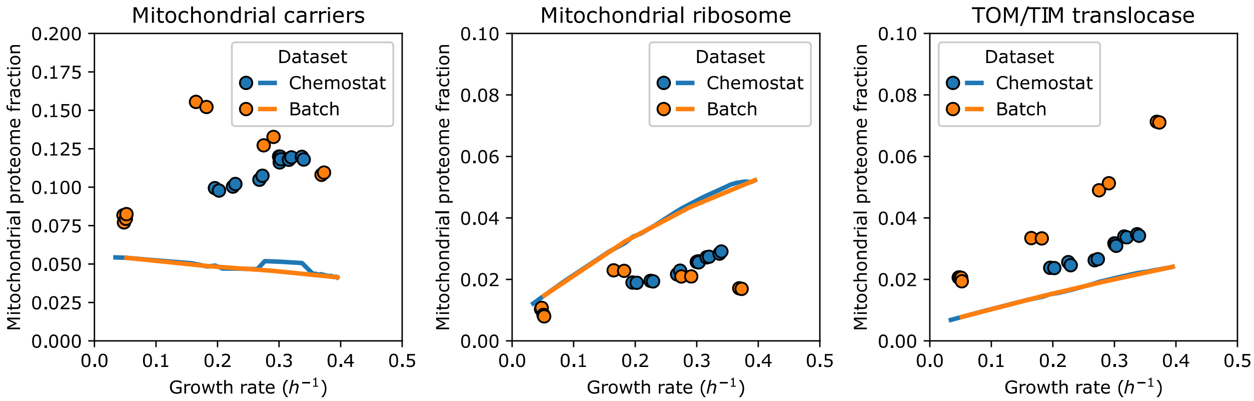

Supplementary Figure 6. Comparison of experimentally determined (circles) and predicted (lines) *mitochondrial* proteome fractions (*i.e.* protein per total mitochondrial protein) occupied by proteins in pathways, located in mitochondria and mitochondrial membrane. Pathway-protein mappings (Table S1) were manually curated, using the reference proteome of *S. cerevisiae* from the UniProt database.

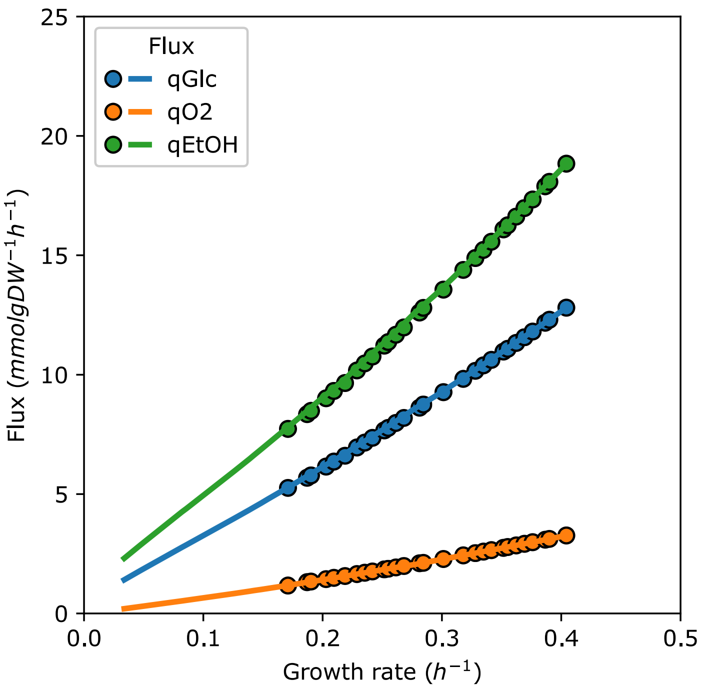

Supplementary Figure 7. Predicted metabolic fluxes for dose-dependent mCherry overexpression in glucose minimal medium (simulation performed analogously to the simulation of mCherry overexpression in SD medium, Fig. 1D, points) and inhibition of translation (lines). The overexpression of mCherry was simulated by setting the desired proteome mass fraction, which mCherry should occupy and running the binary search algorithm to determine the highest growth rate which would still result in a feasible linear program. For inhibition of ribosomes, same procedure was applied, but instead of pre-setting the level of gratuitous protein expression, the activity factor of ribosomes ($0 < f_{Ribo}^{A} \leq 1$) was varied (see Ribosome Capacity Constraint 1).

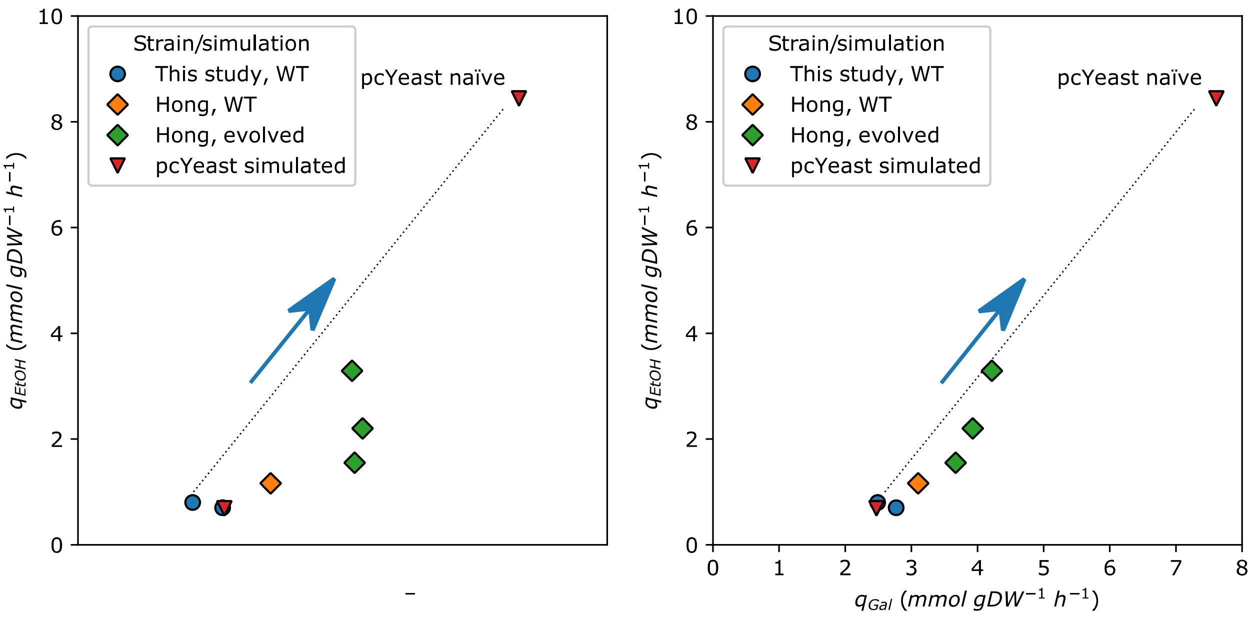

Supplementary Figure 8. Comparison of the physiology of wild-type CEN.PK113-7D and galactose-evolved strains[3] of S. cerevisiae and pcYeast simulations. Left, relationship between specific growth rate and ethanol formation; right, relationship between consumption of galactose and ethanol formation. The "naïve" simulation of pcYeast corresponds to the maximal achievable growth rate on galactose without parameter tweaking (see Supplementary Notes for more details).

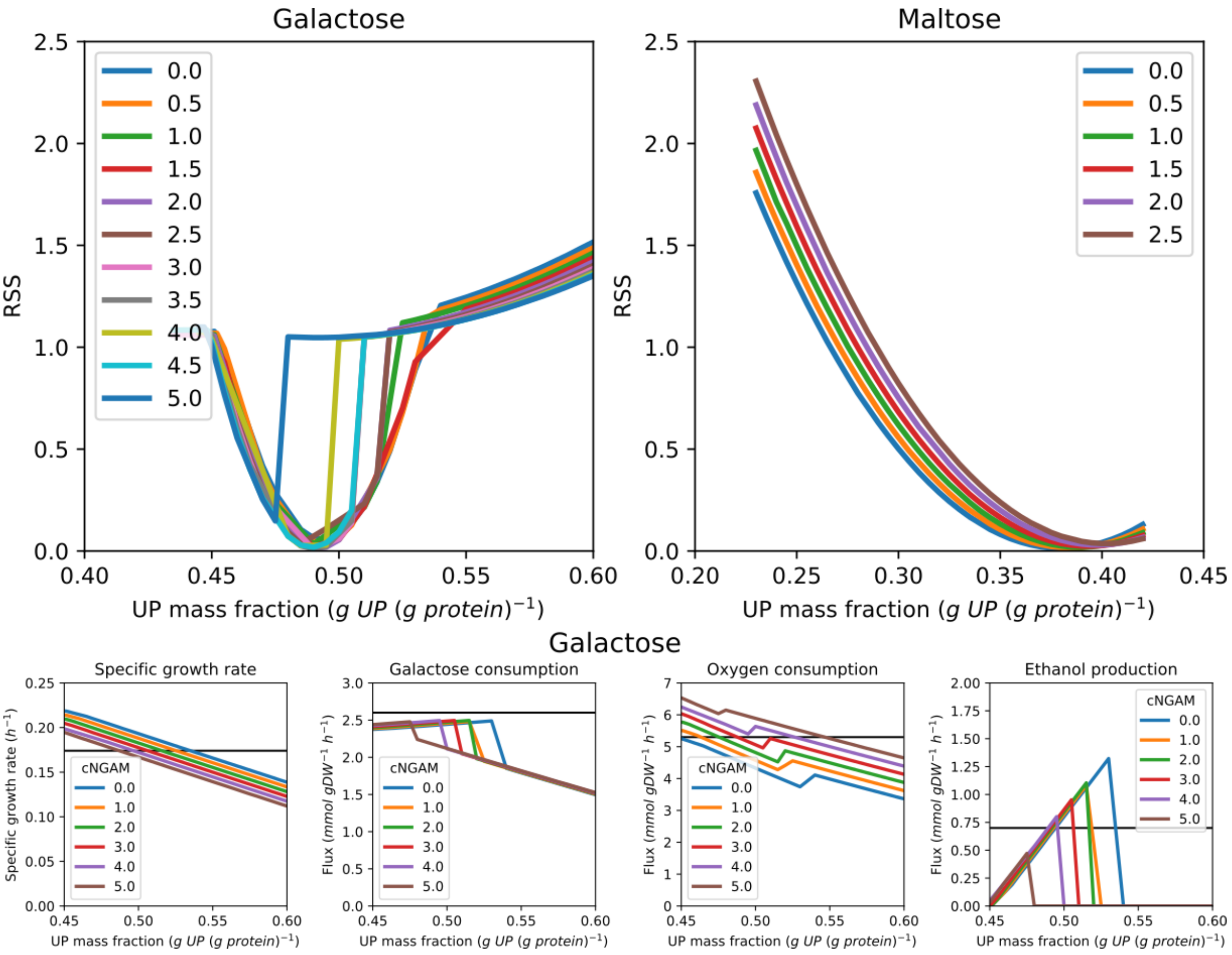

Supplementary Figure 9. Estimation of the parameter values which best represent the experimentally measured physiology of galactose- and maltose-excess batch cultures. The parameters scanned are the minimal UP fraction together with the carbon source-associated NGAM (cNGAM) (upper panels). Prediction of the specific growth rate and metabolic fluxes for different combinations of parameters for galactose are provided in the bottom panels. Details of the procedure used to estimate the parameters are provided in Supplementary Notes.

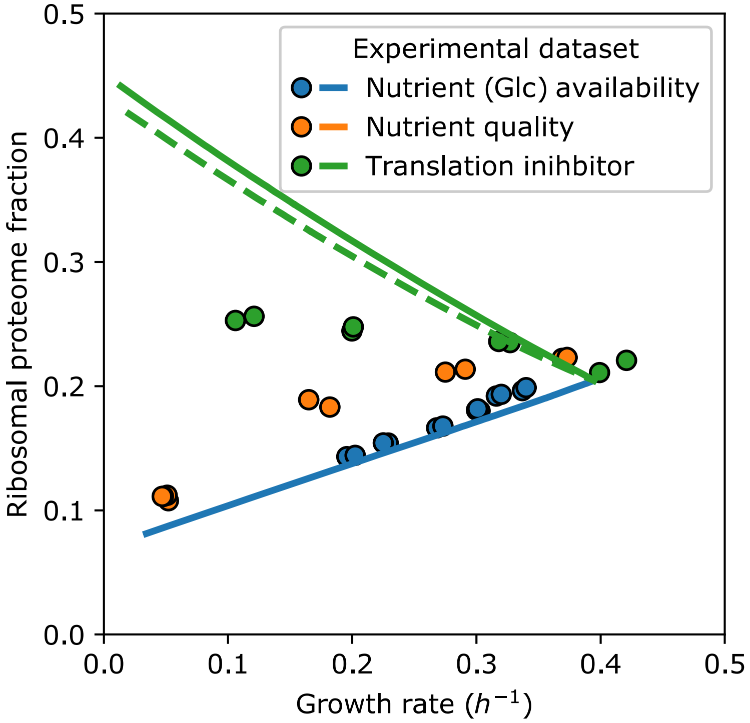

Supplementary Figure 10. Ribosomal proteome fractions (g r-protein/g protein) in different growth scenarios. The dashed line for the simulation of translation inhibition with cycloheximide represents the case where the fraction of inactive ribosomes ϕ_R^0 (see Main Text for details) was linked to the factor of ribosome inhibition: $\phi_R^0 = \phi_R^0(non - inhibited) \times f_{Ribo}^A$, where $f_{Ribo}^A = 1 - f_{Ribo}^{Inh}$. Lines are model predictions, symbols are experimental data points.

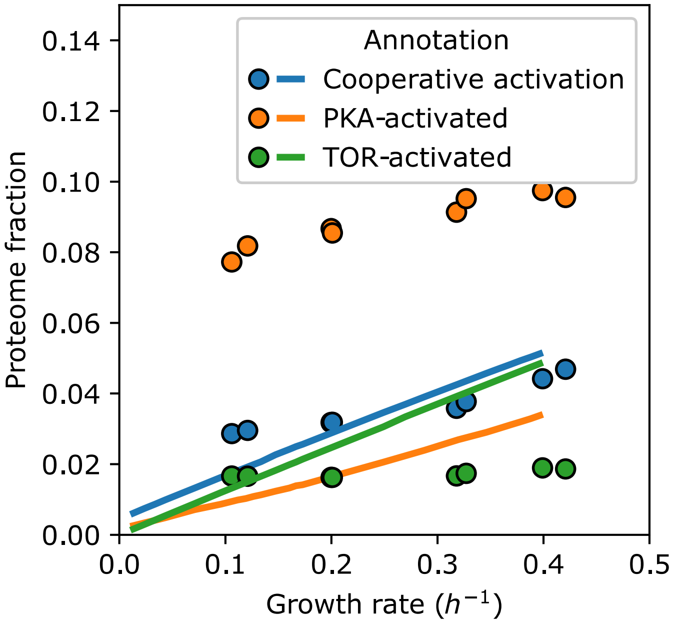

Supplementary Figure 11. Experimentally determined (circles) and predicted (lines) proteome fractions of proteins that were activated either by TOR, by PKA, or cooperatively activated by both TOR and PKA signalling[4]. Groups of specifically activated genes were identified by considering the expression coefficients computed for *S. cerevisiae*, transferred from glycerol- to glucose-rich media containing inhibitors of either PKA, TOR, or both, 20 minutes post-transfer. We selected the genes, for which the expression component was ≥ 2 (Table S2).

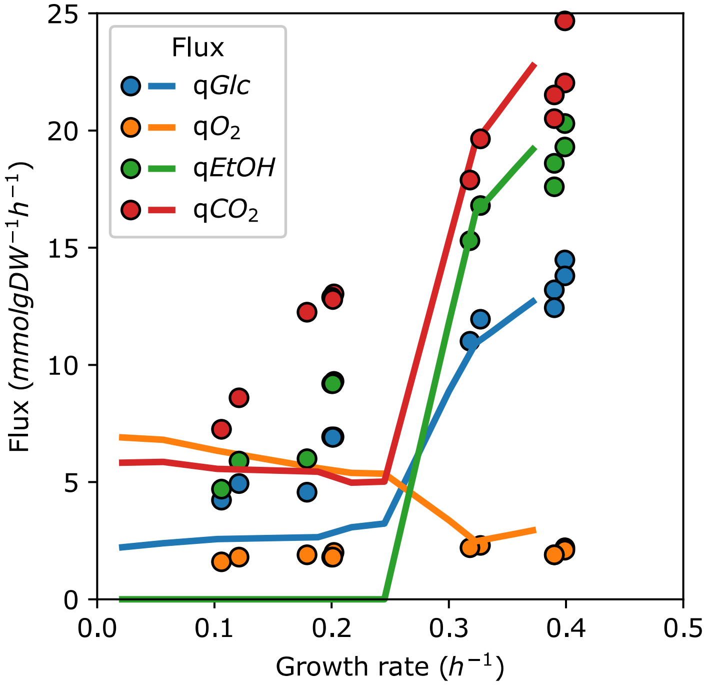

Supplementary Figure 12. The effect of translation inhibition by cycloheximide on the main catabolic fluxes in controlled aerobic batch fermentations on glucose with a phenomenological constraint on the ribosomal fraction. Lines are model predictions with a forced constraint on the maximal ribosomal proteome fraction of $0.25\ g\ r\ protein/\ g\ protein$, symbols are experimental data points.

Supplementary Notes: Generation and Formulation of the pcYeast

Contents

Supplementary Note 1. Formulation of the Linear Program (LP) ... 15
Supplementary Note 2. Model Constraints and Implementation ... 16
2.1 Mass balance constraints ... 16
2.2 Enzyme capacity constraints ... 16
2.3 Ribosome, chaperone and protease constraints ... 18
2.4 Compartment-specific proteome constraints ... 24
2.5 Growth-related constraints ... 30
Supplementary Note 3. Manual curation of turnover values ... 35
3.1 Maltose-specific metabolism ... 35
3.2 Other metabolic enzymes ... 35
Supplementary Note 4. Identification of active Elementary Flux Modes ... 38
Supplementary Note 5. Growth on galactose and maltose ... 41
Supplementary Note 6. Evaluating idle ribosome content in steady-state ... 47

Supplementary Note 1. Formulation of the Linear Program (LP)

$\min v_{Dummy}$, subject to:

1. **Mass balance constraints**:
   $S \times \mathbf{v} = 0,$
   where S is the stoichiometry matrix connecting all metabolites to reactions, and $\mathbf{v}$ is the flux vector for all reactions.
2. **Fixed growth rate at every iteration**: $\mu = \mu_{set}$.
3. **Enzyme capacity constraints**:
   $v_i = k_{cat,i}\,[e_i]\,f_i,\ i \in metabolic\ enzymes$
   where $[e_i]$ is the concentration of a metabolic enzyme e_i, $k_{cat,i}$ is the catalytic rate of enzyme e_i, and f_i is the saturation function of the enzyme e_i. Normally, the saturation level depends on enzyme kinetics and metabolite concentration information. We fix the saturation factor $f_i = 1$ for all enzymes except for hexose transporters and thus compute the minimal proteome requirement to sustain the metabolic fluxes.
4. **Protein synthesis, folding and degradation constraints**:
   $\sum_{i \in cytosolic\ ribosome} v_{syn,i} = k_{cat,cytosolic\ ribosome}\ [Ribosomes]$,
   $\sum_{i \in mitochondrial\ ribosome} v_{syn,i} = k_{cat,mitochondrial\ ribosome}\ [Mito\ ribosomes]$,
   $\sum_{i \in folded\ by\ j} v_{folding,i} = k_{folding,j}\ [j]\ ,\ j \in chaperones,$
   $\sum_{i \in degraded\ by\ j} v_{degradation,i} = k_{degradation,j}\ [j]\ ,\ j \in proteases,$
   These constraints link the protein life-cycle processes to the availability of appropriate components.
5. **Compartment-specific proteome constraints**:
   $\sum v_{syn,i}\, w_i \leq V_{compartment}$ or $\sum v_{syn,i}\, w_i \leq A_{compartment},\ i \in compartment,$
   which limit the proteome according to the volume or area of the appropriate compartment.
6. **Total proteome constraint**:
   $\sum v_{syn,i}\, w_i = P(\mu),\ i \in proteins$
   defining growth rate-dependent protein content of the dry weight of cells (in $g\ protein/gDW$). Note that this is an equality constraint, and has to be fulfilled also at low growth rates, where metabolic fluxes and corresponding protein demands are low. We use an unspecified protein UP with average protein composition to maintain this equality and thus protein density.
7. **Growth-related metabolic reactions**:
   $v_i = k \times \mu$
   Examples of such reactions are the growth-associated ATP maintenance (GAM), which accounts for the ATP expenditures coupled to growth that are not explicitly modeled. The biomass reaction describes the demand for all biomass components (except proteins, which are explicitly modeled as described above), proportional to the growth rate.

Supplementary Note 2. Model Constraints and Implementation

We converted the linear program of our model to CPLEX LP format (.lp) and used the SoPlex solver[5] to solve the problem with high precision (maximal feasibility and optimality violation: 10^{-12}). The advantage of writing our model as CPLEX LP format is the ability to add constraints directly without any restrictions imposed by the software which is used to handle genome-scale models. Additionally, most solvers read CPLEX LP format, so we are not restricted to the solvers offered by RAVEN[6], COBRA[7] or PySCeS CBMPy[8] packages. The maximal feasible growth rate μ_{max} is determined by using binary search, i.e. running LPs to test for feasibility in a predefined range of growth rates to find the highest feasible value (see Section 1). We consider the simulated growth rate to be the highest feasible (μ_{max}) if a very small increment in growth rate $\Delta\mu$ results in an infeasible linear program (i.e. at $\mu = \mu_{max} + \Delta\mu$) . We set $\Delta\mu \leq 10^{-4}\ h^{-1}$ in our simulations. Note that the model implementation is available in two formats, based in either MATLAB or Python, and the underlying logic/assumptions are valid for both implementations.

The model introduces novel proteome constraints which are in essence phenomenological limits based on observation on the CEN.PK113-7D strain of *S. cerevisiae*, rather than being hard physico-chemical constraints. In the next subsections, we describe every constraint in the model in more detail.

2.1 Mass balance constraints

Mass Balance Constraint 1:

The first constraint in our model is the steady-state condition ($S \times \mathbf{v} = 0$), where $\mathbf{v}$ is the flux vector and S is the stoichiometric matrix of $i \times j$ columns (reactions) and rows (metabolites), respectively. For any reversible reaction in the Yeast7.6 model, we split them into two unidirectional reactions, forward and reverse. This constraint is the main constraint in stoichiometric models, and is applies to all molecular species in the cell. In the constraint expressions below, we use the convention of v_i for a flux.

2.2 Enzyme capacity constraints

Flux Capacity Constraint 1: Metabolic coupling

For all metabolic reactions $\{v_1, v_2, \dots, v_n\} \in metabolic\ rxns$ that are catalyzed by enzymes (with enzyme concentrations $[e_i]$), the model now has a demand of producing metabolic enzymes. Moreover, every enzyme e_i is diluted by growth and/or degraded towards individual amino acids. In order not to violate the steady state of the system, the sum of dilution and degradation fluxes must be equal to that of protein synthesis. Thus we define the steady-state enzyme concentration as follows:

$$[e_i] = \frac{v_{syn,i}}{\mu + k_{deg}} \quad (1)$$

where k_{deg} is the protein degradation rate. Assuming the enzyme works at its maximal reaction rate, i.e. $v = v_{max}$, we determine the relationship between metabolic fluxes and enzyme demand [9]:

$$[e_i] = \frac{v_{max}}{k_{cat,i}} \tag{2}$$

where $k_{cat,i}$ is the turnover constant for the enzyme e_i.

We fixed the k_{deg} for all peptides in the model to the median protein degradation rate $k_{deg} = 0.043\ h^{-1}$, according to experimental measurements of protein stability at $\mu = 0.1\ h^{-1}$ [10]. To establish the ratio between fluxes through metabolic reactions and enzyme demands, we acquired $k_{cat,i}$ values using the GECKO toolbox[11], as well as manually curated values for enzymes in central carbon metabolism[12], or other sources, such as BRENDA[13] or SABIO-RK[14].

In the model, the costs for both monomeric enzymes, as well as their complexes, are associated with complex assembly reactions. Enzyme complexes are formed based on the known stoichiometric ratios of the individual subunits:

$$R_{complex\ formation,i}\text{:} \sum_{k \in cplx_i} s_k e_k \rightarrow cplx_i \tag{3}$$

where s_k is the stoichiometric coefficient of the subunit e_k. We collected information about stoichiometry of the complexes from UniProt[15], MetaCyc[16], and Protein Data Bank[17]. For each complex of metabolic enzymes, its demand is considered to be the sum of costs for individual reactions, using this enzymatic complex:

$$\sum_{j=1}^{m} \frac{v_j}{k_{cat,i,j}} \leq \frac{v_{complex\ formation,i}}{\mu + k_{deg}} \tag{4}$$

where $k_{cat,i,j}$ is the turnover constant of the complex i, catalyzing the j-th reaction.

Protein Balance Constraint 2a: Dilution by growth

All proteins e_i are diluted by growth, i.e. with a rate constant equal to the growth rate μ. Therefore, we set the flux through the dilution reaction of the complex $cplx$ to $\mu \times [e_i]$:

$$R_{dil,i}\text{:}\ cplx_i \rightarrow \emptyset \text{ and} \tag{5}$$

$$v_{dil,i} - \mu \frac{v_{complex\ formation,i}}{\mu + k_{deg}} = 0 \tag{6}$$

Protein Balance Constraint 2b: Degradation to free amino acids

Similarly, we set the flux through degradation reaction of the complex $cplx$ to $k_{deg} \times [e_i]$.

$$R_{deg,i}: cplx_i + mn\ ATP + (m + 2n - 1)\ H_2O \rightarrow \sum_{j \in aa} kj + mn\ ADP + mn\ P_i + mn\ H^+, \sum j = n \text{ and}$$

(7)

$$v_{deg,i} - \ k_{deg} \frac{v_{complex\ formation,i}}{\mu + k_{deg}} = 0$$

(8)

2.3 Ribosome, chaperone and protease constraints

Ribosome Capacity Constraint 1: Capacity of the cytosolic ribosome

We considered the ribosome demand to be affected by the processes of ribosome scanning, translation initiation, elongation and termination. We implemented expenditures of resources for the processes outside translation elongation as respective increments to the length of the amino acid chain. We constructed a coupling constraint for the synthesis reaction of peptide species e_i as follows: the demand of ribosomes for synthesis of e_i (denoted as $Ribo_{e_i}$) is dependent on peptide elongation rate $k_{cat,ribo}$, as well as on the length of the peptide $l_{peptide}$ (i.e. translation elongation), together with the increments of l_i and $l_{scanning}$ for translation initiation, number of scanning steps through the 5'UTR region of the transcript for the protein e_i and translation termination (an equivalent of one amino acid), respectively. For each peptide species e_i, the constraint is defined as:

$$v_{syn,e_i}\,[mmol/gDW/h] = \frac{k_{cat,ribo}\ [aa/h]}{l_{i,e_i} + \ l_{scanning,e_i} + l_{peptide,e_i}\ [aa] + 1} Ribo_{e_i}\,[mmol/gDW]$$

(9)

where $k_{cat,ribo}$ is the maximal peptide elongation rate, $k_{cat,ribo} = 10.5\ aa\ s^{-1} = 3.78 \times 10^4\ aa\ h^{-1}$ [18].

Section 6 describes in more detail how we also adjust ribosomal protein synthesis according to estimation that ca. 8% of yeast proteome is occupied by ribosomal proteins at zero growth[18]. Here we provide only the description of how we accounted for this in the model. We defined the extra demand for the ribosomal protein allocation by the following expression:

$$Ribo_{nontranslating} = \phi_R^0 \times f_{P,biomass}(\mu)[g\ protein/gDW]/M_{P,ribo}[g\ protein/mmol]$$

(10)

where $\phi_R^0 = 0.053$ is the proteome fraction, always filled with non-translating ribosomes (see Section 6) and $M_{P,ribo} = 1388.488\ g/mmol$ is the molar mass of the protein component of the fully assembled ribosome. Following[9], we define that $Ribo_{e_i}$ is the amount of ribosomes that translates e_i and thus the total ribosome abundance in the model equals $\sum_{e_i} Ribo_{e_i} + Ribo_{nontranslating} = [Ribosome]$.

We modulated the catalytic activity of all protein translation machinery (i.e. ribosomes, ribosome assembly factors, as well as translation initiation, elongation and termination factors), by the ribosome

activity factor, which is $f_{Ribo}^{A} = 1$ for unperturbed cells. In case of inhibition of ribosomal activity (e.g. cycloheximide treatment), the $1 - f_{Ribo}^{Inh} = f_{Ribo}^{A} < 1$.

The balance between fluxes through ribosome biosynthesis, dilution, and degradation reactions is derived in the same manner as for the metabolic reactions (see Constraints 1, 2a and 2b in Section 2.2). By using the relationship $\sum_{e_i} Ribo_{e_i} + Ribo_{non-translating} = [Ribosome]$ for summing up the individual amounts of ribosomes, synthesizing every single peptide species to the total ribosomal pool, we set the requirement for ribosome synthesis (assembly of ribosomal units) as follows:

$$[Ribosome] \leq f_{Ribo}^{A} \times \frac{v_{complex\ formation,ribo}}{\mu + k_{deg,ribo}} \tag{11}$$

where $v_{complex\ formation,ribo}$ is the ribosome complex formation (assembly) reaction, for which we used annotations for the subunits of cytosolic ribosome from [19].

Ribosome Capacity Constraint 2: Cytosolic translation initiation factors

Translation initiation in yeast has been studied in great detail to reveal the mechanism of translation initiation in eukaryotic cells[20]. A number of translation initiation factors ($eIF1A$, $eIF1B$, $eIF2A$, $eIF1B$, $eIF3$, $eIF4A$, $eIF4B$, $eIF4E$, $eIF4G$, $eIF5$) participate in this process.

Some of the initiation factors also form separate complexes which scan the 5'UTR region until finding the first codon AUG to form the 48S ribosomal complex. Thus the 5'UTR length greatly influences the cost of the scanning process in translation initiation. To determine the TSS (translation starting site i.e. number of nucleotides before the coding sequence starts) for each gene, we used SMORE-seq data[21]**.** We compared the SMORE-seq data with the yeast genome sequence R64-1-1 from the SGD database[22] to determine the start and end position of the CDS. For a gene with no experimental estimation of TSS, we assume its 5'UTR length to be 50 nucleotides (median of 5'UTR length). For a gene that has TSS inside the CDS, we assume its 5'UTR length to be zero. This information is then used to compute the mRNA-specific costs of translation initiation.

To describe the demand for translation initiation factors, we defined all initiation translation factors as a single complex (TIF). One of the increments to the expenses to the protein synthesis, as described before, is the time for the ribosome to scan along the mRNA to find the first coding triplet. [23] proposed a random walk algorithm to estimate the number of steps in the 5'UTR region ($l_{scanning}$) (see Ribosome Capacity Constraint 1). The complex hydrolyzes one ATP per each scanning step.

Here, we assumed that a ribosome scans transcripts at the rate of about 10 nucleotides per second ($k_{scan} = 10\ nt\ s^{-1} = 3.6 \times 10^4\ nt\ h^{-1}$), which is close to the maximal peptide elongation rate of $k_{cat,ribo} = 10.5\ aa\ s^{-1}$. Thus, from the perspective of ribosomal demand, we considered one scanning step at the 5'UTR region as one additional amino acid and added the number of scanning steps ($l_{scanning}$)

to the total protein length. For each peptide species e_i we define the relationship between translation of protein species e_i and the demand of TIF as follows:

$$v_{syn,e_i}\,[mmol/gDW/h] - \frac{k_{scan}[nt\ h^{-1}]}{l_{scanning,i}\ [nt]} TIF_{e_i}[mmol/gDW/h] = 0$$

(12)

where v_{syn,e_i} is a flux through the synthesis reaction of e_i, k_{scan} is the ribosome scanning rate and TIF_{e_i} is the amount of TIF required for initiation of synthesis of peptide e_i.

Alike the ribosomal amounts for each peptide species e_i, the amounts of TIF could be summed up to determine the total pool of TIF: $\sum_{e_i} TIF_{e_i} = [Translation\ initiation\ factors]$. We thus set the requirement for the synthesis of TIF (assembly of translation initiation factors into the TIF complex) as follows:

$$[Translation\ initiation\ factors] \leq f_{Ribo}^{A} \times \frac{v_{complex\ formation,TIF}}{\mu + k_{deg,TIF}}$$

(13)

Ribosome Capacity Constraint 3: Cytosolic translation elongation factors

We also accounted for the demand of translation factors $eEF1A$, , $eEF1B$, $eEF2$, $eEF3$ [24–26], as well as the hydrolysis of the nucleotide triphosphates during peptide translation. In total, 2 GTP and 2 ATP are spent for elongation of the peptide chain by one amino acid.

The elongation factors are required to catalyze their reactions at the speed equivalent to the peptide elongation, thus we assumed $k_{cat,EF} = k_{cat,ribo}$. To couple the elongation translation factor (i.e., $\{eEF1A, eEF1B, eEF2, eEF3\}$) biosynthesis reactions with the demand of these factors in translation of peptides, we considered that synthesis of each protein e_i requires each of the eEF_j (eEF_{j,e_i}). For each peptide species e_i and eEF_j, the constraint was defined as follows:

$$v_{syn,e_i} - \frac{k_{cat,EF}}{l_{peptide,e_i}}\, eEF_{j,e_i} = 0$$

(14)

where v_{syn,e_i} is a flux through protein synthesis reaction of e_i and $l_{peptide,e_i}$ is the length of protein e_i. Alike the ribosome demand for each peptide species e_i, the amounts of eEF_{j,e_i} could be summed up to determine the total pool of eEF_j:

$$\sum_{e_i} eEF_{j,e_i} = [eEF_j]$$

(15)

By using this relationship, we set the requirement for the complex formation of eEF_j as follows:

$$[eEF_j] \leq f_{Ribo}^{A} \times \frac{v_{complex\ formation,eEF_j}}{\mu + k_{deg,eEF_j}}$$

$$ (16) $$

Ribosome Capacity Constraint 4: Cytosolic translation termination factors

We adopted a termination model proposed by [24]. In this model, the translation termination factor ($eRF1$) binds with GTP-bound form of $eRF3$. $eRF3 - GTP$ is a ribosome-binding factor that recognizes the stop codon. Thus, translation termination requires one molecule of GTP.

To also account for the cost of translation termination factors, we set the demand of eRF ($eRF_{\ e_i}$) for each protein synthesis reaction. We considered the termination rate to be equal to $k_{cat,eRF} = 0.15\ s^{-1}$ [27]. Thus we formulated the constraint as follows:

$$ v_{syn,e_i} - k_{cat,\mathrm{eRF}}\ \mathrm{eRF}_{\ e_i} = 0 \tag{17} $$

where v_{syn,e_i} is a flux through protein synthesis reaction of e_i and $k_{cat,\mathrm{eRF},}$ is the turnover rate of $eRF2$ binding. Alike the ribosomal amounts for each peptide species e_i, the amounts of eRF could be summed up to determine the total pool of eRF:

$$ \sum_{e_i} eRF_{\ e_i} = [eRF] \tag{18} $$

By using this relationship, we set the requirement for the complex formation of eRF as follows:

$$ [eRF] \le f_{Ribo}^{A} \times \frac{v_{complex\ formation,eRF}}{\mu + k_{deg,eRF}} \tag{19} $$

Ribosome Capacity Constraint 5: Cytosolic ribosome assembly factors

Ribosome assembly factors are required for assembling new ribosome from its subunits. Assembly factors facilitate assembly of ribosomal subunits into supercomplexes, which are transported from nucleus to cytoplasm. After that, the ribosomal subunits go through maturation. We used our proteomics data of sugar-excess cultures in order to estimate the turnover number of the ribosome assembly factors, so the predicted demand of these factors would match the experimentally determined abundance. Following that, we set the $k_{cat,ribo\ assembly} = 1\ min^{-1}$.

As there are many species of ribosome assembly factors, we consider them as a single complex and assume that all ribosomal assembly factors are bound to ribosomal subunits[28]. We thus introduced a coupling constraint, setting the demand for ribosome assembly factors $v_{complex\ formation,ribo\ assembly}$ to be proportional to the ribosome assembly reaction $v_{complex\ formation,ribo}$ as follows:

$$ v_{complex\ formation,ribo} \le f_{Ribo}^{A} \times k_{cat,ribo\ assembly} \frac{v_{complex\ formation,ribo\ assembly}}{\mu + k_{deg,ribo\ assembly}} \tag{20} $$

Ribosome Capacity Constraint 6: Mitochondrial ribosome capacity

The mitochondrial ribosome translates the peptides for 7 proteins of oxidative phosphorylation system and one subunit of the mitochondrial ribosome. Due to this fact, we provided a simplified description of the mitochondrial ribosome in the model, i.e. we did not take into account the expenses of translation initiation, termination and/or associated factors. Even though, we modelled the translation elongation in a similar fashion as for the cytosolic ribosome (Ribosome Capacity Constraint 1). With defining the demand for the mitochondrial ribosome $MRibo_{e_i}$ for the synthesis of the peptide e_i with amino acid chain length $l_{peptide,e_i}$, we formulated the following constraint:

$$v_{syn,mito,e_i} = \frac{k_{cat,ribo,mito}}{l_{peptide,e_i}} MRibo_{e_i} \tag{21}$$

where the elongation rate of the mitochondrial ribosome was estimated to be $k_{cat,ribo,mito} = 2.7\ aa\ s^{-1}$, according to the proteomics measurements in glucose-limited chemostat cultures.

By using the relationship $\sum_{e_i} MRibo_{e_i} = [mitoRibosome]$ between the amount of mitochondrial ribosomes, synthesizing single peptide species and total pool of mitochondrial ribosomes, we set the requirement for ribosome synthesis (assembly of ribosomal units) as follows:

$$[mitoRibosome] \leq \frac{v_{complex\ formation,mitoribo}}{\mu + k_{deg,mitoribo}} \tag{22}$$

where $v_{complex\ formation,mitoribo}$ is the mitochondrial ribosome complex formation (assembly) reaction, for which we used annotations for the subunits of mitochondrial ribosomal subunits from [29,30].

Proteome Maintenance Constraint 1: Folding of the proteins

Chaperone-mediated folding was considered for the cytosolic proteins by multiple chaperone systems. We acquired the information on the clients of these chaperones from the chaperon atlas[31]. For the cytosolic proteins without a chaperone specified, we assumed folding by chaperone SSB, one of the most abundant and well-studied chaperones. All the proteins in the mitochondria were assumed to be folded by mitochondrial isoform (mt) of Hsp70.

Thus, for each chaperone, the flux through its complex formation reaction could be computed from the sum of the fluxes through protein folding reactions for all its clients:

$$\sum_{e_i} \frac{1}{m} v_{folding,i} \leq k_{cat,chaperone} \frac{v_{complex\ formation,chaperone}}{\mu + k_{deg,chaperone}} \tag{23}$$

where m is a number of chaperones, known to fold the same client and $k_{cat,chaperone} = 0.95\ min^{-1}$ is a turnover constant, reported from ATP turnover measurements for SSB[32]. Turnover values of the same order were also obtained for chaperone BiP[33].

Proteome Maintenance Constraint 2: Degradation of the proteins

For most of the model proteins, protein degradation is described as a proteasome-dependent process. However, the 8 proteins produced by mitochondrial ribosome are assumed to be degraded by a mitochondrial protease PIM1 instead. All protein degradation reactions were coupled with the complex formation of the proteases (either proteasome complex or PIM1) using the following constraint:

$$\sum_{e_i} v_{deg,e_i} \leq k_{cat,protease} \frac{v_{complex\,formation,protease}}{\mu + k_{deg,protease}}$$

(24)

where $k_{cat,\mathrm{protease}}$ is the catalytic turnover rate of the protease. The proteasome (subunit composition reported in [34]) can degrade proteins with a turnover rate of $k_{cat,proteasome} = 2.3\ min^{-1}$ [35], while for PIM1, we used the value reported for its homolog in *E. coli*, Lon protease: $k_{cat,Lon} = 0.26\ s^{-1}$ [36]. For both complexes, we set the cost of protein degradation to be 1.35 molecules of ATP per amino acid[37].

Proteome Maintenance Constraint 3: Protein transport to mitochondria

Proteins are transported to mitochondria by the TOM-TIM translocase system[38]. TOM complex is located in the outer mitochondrial membrane, while TIM22 and TIM23 are located in the inner membrane. All the proteins first enter the mitochondrial intermembrane space through TOM. Next, proteins of the inner mitochondrial membrane (IMM) and mitochondrial matrix are transported into the matrix via TIM23. Then, the IMM proteins are inserted from the matrix side by TIM22. Thus we define a set of mitochondrial transporters $MT = \{TOM, TIM22, TIM23\}$ and using the annotations of the localization of these proteins, define which transporters (or combinations of these) are used for which transport processes.

$$\sum_{e_i} v_{transport,e_i,MT_j} \leq k_{cat,MT_j} \frac{v_{complex\,formation,MT_j}}{\mu + k_{deg}}$$

(25)

The two main forces controlling protein transport to and out of the mitochondria are (i) membrane potential and (ii) force generation by mitochondrial chaperones, mainly mtHsp70. [39] reported that a single cycle of mtHsp70 (ATP hydrolysis) creates a power stroke of ca. 3.5 nm (10 residues of the unfolded protein chain), we set additional requirement for ATP hydrolysis in mitochondria:

$$mtHsp70\ ATPase\ (ATP_{mtHsp70}): ATP + H_2O \rightarrow ADP + P_i + H^+$$

(26)

$$\sum_{e_i} \frac{l_{e_i}\ [aa]}{10\ [aa\ stroke^{-1}]}\ v_{transport,e_i,TIM23} = ATP_{mtHsp70}$$

(27)

Proteome Maintenance Constraint 4: Balance of complex degradation and dilution by growth

As for the metabolic proteins, we define the same balance of complex formation and their dilution by growth for all the macromolecular complexes (ribosomes, translation factors, chaperones, proteases etc.) in the model:

$$v_{degradation} = k_{deg} \frac{v_{complex\,formation}}{\mu + k_{deg}} \text{ and } v_{dilution} = \mu \frac{v_{complex\,formation}}{\mu + k_{deg}}$$

(28)

2.4 Compartment-specific proteome constraints

Proteome Constraint 1: Total proteome mass constraint

[40] observed that the sum mass of the proteins in dry cell mass for the CEN.PK 113-7D strain is growth rate-dependent. Notably, in the measurements of [40], the protein content and water content were reported separately, but since water is a byproduct of protein polymerization, we added these two components together. We implemented the following constraint, so that at any given growth rate, the protein content would correspond to the experimentally determined protein content in dry cell biomass $f_{p,biomass}(\mu)$.

$$\sum \frac{v_{syn,i}}{\mu + k_{deg}} [mmol/gDW] \times \frac{MW_i \left[\frac{g\ protein}{mol}\right]}{1000 \left[\frac{mmol}{mol}\right]} = f_{p,biomass}(\mu)\ \ [gram\ protein/gDW] \tag{29}$$

where $f_{p,biomass}(\mu) = 0.4854\ \mu + 0.3888$.

Proteome Constraint 2: Cytoplasmic volume constraint

In order to establish a constraint on the cytoplasmic volume, we assumed that all the peptides e_i, which are synthesized by either the cytosolic or mitochondrial ribosome, occupy *total* cytosolic volume regardless of their final compartment of action. For each protein e_i, we estimated volume of a single molecule of protein: [41] assumed that the protein molecule is a sphere and its radius r was assumed to be a function of molecular weight MW:

$$r_i = 0.066\ MW_i^{\frac{1}{3}} \tag{30}$$

We then used r_i to estimate the volume and area occupied by each protein:

$$V_i = \frac{4}{3}\pi r_i^3 \tag{31}$$

We used the data of [42] to estimate the growth-dependent volume $V_{cell}(\mu)$ of single cells of *S. cerevisiae*, which vary from ca. $V_{cell} = 20.5\ \mu m^3$ (at $\mu \to 0$) to $V_{cell} = 32.6\ \mu m^3$ (at $\mu = 0.41\ h^{-1}$). Then we used the data of [40] to estimate the protein-available fraction $f_{pa}(\mu)$ of the total cell volume, so that the sum mass of the proteins would correspond to the protein content in dry cell biomass $f_{p,biomass}(\mu)$, determined for the CEN.PK 113-7D strain[40].

The protein-available fraction $f_{pa}(\mu)$ can be decomposed into two variables, $f_{pa}(\mu) = c \times f_{p,biomass}(\mu)$, where c is a growth rate-independent conversion factor. We can estimate the value of c by using the following example for growth rate $\mu = 0$. For any growth rate, the following is true (see Proteome Constraint 1):

$$\sum \frac{v_{syn,i}}{\mu + k_{deg}} [mmol/gDW] \times \frac{MW_i \left[\frac{g\ protein}{mol}\right]}{1000\ \left[\frac{mmol}{mol}\right]} = f_{p,biomass}(\mu)\ \ [gram\ protein/gDW]$$

(32)

Considering a single molecule of protein YDRUMD1 ($MW_{YDRUMD1} = 48870\ g/mol$), we can estimate both volume and mass of a single molecule of YDRUMD1:

$$r_{YDRUMD1} = 0.066 \times (MW_{YDRUMD1})^{\frac{1}{3}}$$

(33)

$$V_{YDRUMD1} = \frac{4}{3}\pi r_{YDRUMD1}^3 = 58.85\ nm^3$$

(34)

$$m_{YDRUMD1} = MW/N_A = 8.115 \times 10^{-20}\ g$$

(35)

At zero growth $\mu = 0$, the $f_{p,biomass}(0) = 0.3888\ g/gDW$. Considering this mass to be composed solely of YDRUMD1 molecules, we can compute the volume these molecules would occupy:

$$V_{prot} = \frac{V_{YDRUMD1} \times f_{p,biomass}(0)}{m_{YDRUMD1}} = 2.820 \times 10^{11} \mu m^3$$

(36)

[40] also estimated that the volume of 1 gram of dry cell weight is $V_{gDW} = 1.7\ mL/gDW$, which was reported to remain relatively constant given different growth rates. Thus by computing the ratio of the volume, occupied by YDRUMD1, and the total cell volume, we can yield the $f_{pa}(0)$:

$$f_{pa}(0) = \frac{V_{prot}}{V_{gDW}} = 0.1658$$

(37)

Finally, by knowing the $f_{p,biomass}(0)$ and $f_{pa}(0)$, we can compute c:

$$c = \frac{f_{pa}(0)}{f_{p,biomass(0)}} = 0.426$$

(38)

It should be noted that this computation is valid regardless of just single protein species taken, as the relationship between the molecular weight and the volume of the sphere is linear[41], thus any protein would fulfil the requirements.

To estimate the contribution of any protein to the protein-available volume, we used the reasoning of [41] further. For every protein species e_i, we calculated the volumes of single protein molecules in nm^3, and considered the following relationship to acquire the volume in $\mu m^3/mmol$ of protein:

$$V_{molar,i} = V_i \times 10^{-9} \frac{\mu m^3}{nm^3} \times 6.022 \times 10^{20}\ mmol^{-1}$$

(39)

By using the volume of 1 gram of dry cell weight $V_{gDW} = 1.7\ mL/gDW$, we can estimate the number of cells in a gram of cell dry weight:

$$N_{cells} = \frac{V_{gDW} \times 10^{-6} \frac{m^3}{mL}}{V_{cell} \times 10^{-18} \frac{m^3}{\mu m^3}}$$

(40)

and then distribute the molar volume to the number of cells in 1 gram of dry cell weight, yielding how much volume would a 1 $mmol/gDW$ of protein occupy in a single cell:

$$V_{i,single\ cell} = \frac{V_{molar,i}}{N_{cells}}$$

(41)

Then we formulate the constraint as follows:

$$\sum \frac{v_{syn,i}}{\mu + k_{deg}} \times V_{i,single\ cell} = V_{cell}(\mu) \times f_{pa}(\mu)$$

(42)

Proteome Constraint 3: Mitochondrial volume constraint

Analogously to the all the total cytosolic volume constraint, we formulated a mitochondrial protein volume constraint, which constrains the sum volume of all model proteins allocated to this compartment (including mitochondrial ribosomes and import machinery). We formulated the following constraint:

$$\sum_{j \in Mito} \frac{v_{syn,j}}{\mu + k_{deg}} \times V_{j,single\ cell} = V_{cell}(\mu) \times f_{pa}(\mu) \times f_{ma}$$

(43)

The mitochondrial proteome volume is always expressed as a fixed fraction f_{ma} (for "mitochondrial available") of the total volume available to the proteins $f_{pa}(\mu)$. We set this fraction $f_{ma} = 0.17$, which sets the available volume of mitochondrial proteins ($c \times f_{ma}$) to about 7.25% of the total protein-available cellular volume, which is close to the 7.4%[43].

Proteome Constraint 4: Inner mitochondrial membrane constraint

Furthermore, all inner mitochondrial membrane (IMM) proteins from the metabolic model (e.g. electron transport chain proteins, protein translocation complexes and transporters for metabolites) were used to formulate a constraint on IMM surface area. Since the defining factor in this case is the area of the cross-section of these proteins, which spans the membrane (and for globular proteins is defined as $A_i = \pi r_i^2$), we modified the previously derived relation to account for the IMM surface area:

$$A_{molar,i} = A_i \times 10^{-6} \frac{\mu m^2}{nm^2} \times 6.022 \times 10^{20}\ mmol^{-1}$$

(44)

and then distribute the molar area to the number of cells in 1 gram of dry cell weight (see Proteome Constraint 1), yielding how much IMM surface area would a $1\ mmol/gDW$ of protein occupy in a single cell:

$$A_{i,single\ cell} = \frac{A_{molar,i}}{N_{cells}} \tag{45}$$

And formulated the constraint as follows:

$$\sum_{j \in IMM} \frac{v_{syn,j}}{\mu + k_{deg}} \times A_{j,single\ cell} \leq A_{IMM} \tag{46}$$

[44] and [45] reported that the surface area of the mitochondrial inner membrane is about $15\ \mu m^2$ in rich YPD medium. We used these measurements to estimate of the maximal mitochondrial membrane area.

Compared to $D = 0.27\ h^{-1}$ in glucose limited chemostats, where maximal mitochondrial protein expression is the highest, the growth rate on YPD-glucose is about $0.45\ h^{-1}$, and $0.4\ h^{-1}$ on minimal glucose medium. Therefore, if we extrapolate the drop in mitochondrial proteins (Figure 4d in the main text), then the mitochondrial content on YPD must be lower than on minimal media in batch. As shown experimentally[46], the fraction of the cell volume, occupied by mitochondria, can differ up to 6-fold between cells grown in glucose-rich medium (low number of mitochondria) and ethanol-growing (high number of mitochondria) cells. Then the 6 times decrease in mitochondrial protein content on YPD reported in literature is consistent with our proteomics data that show that maximal mitochondrial protein expression (glucose-limited chemostats, $D = 0.27\ h^{-1}$) is about 4 times higher than on glucose minimal media in batch ($\mu = 0.4\ h^{-1}$).

Assuming that mitochondrial inner membrane area-to-volume ratio is constant, in this extreme case, a 6-fold increase in volume fraction would correspond to a 6-fold increase in mitochondrial inner membrane surface area, setting the upper bound to ca. $90\ \mu m^2$. As [47] reported that proteins occupy about 80% of the mitochondrial inner membrane area, we can account for that by reducing the upper bound by 20%, to $72\ \mu m^2$. In the end, we set the upper bound of surface area of the inner mitochondrial membrane to $75\ \mu m^2$ in our model.

Proteome Constraint 5: Plasma membrane constraint

We previously used data from [42] to determine the volume of a single cell. Assuming a cell of *S. cerevisiae* to be sphere-shaped, we can estimate the plasma membrane surface area using the volume of the cell:

$$A_{PM} = 4 \times \pi \times r^2 \tag{47}$$

where $r = \left(\frac{3}{4\pi} V_{cell}\right)^{\frac{1}{3}}$, a radius of a spherical cell.

To estimate the surface area the cross-section of these proteins occupy, we used the previously derived relation (Proteome Constraint 4) to account for the plasma membrane surface area:

$$A_{molar,i} = A_i \times 10^{-6} \frac{\mu m^2}{nm^2} \times 6.022 \times 10^{20}\ mmol^{-1}$$

(48)

and then distribute the molar area to the number of cells in 1 gram of dry cell weight (see Proteome Constraint 1), yielding how much PM surface area would a 1 $mmol/gDW$ of protein occupy in a single cell:

$$A_{i,single\ cell} = \frac{A_{molar,i}}{N_{cells}}$$

(49)

And formulated the constraint as follows:

$$\sum_{j \in PM} \frac{v_{syn,j}}{\mu + k_{deg}} \times A_{j,single\ cell} \leq A_{PM}$$

(50)

Proteome Constraint 6: Glucose transport membrane area

During model development, it became clear that at low saturation of the glucose transporter, mimicking low dilution rates of glucose-limited chemostat cultures, we overestimated growth rate due to all the membrane being predicted to be occupied with glucose transporters. In chemostat cultures, [48] measured glucose transport kinetics at different dilution rates and concluded that the maximal rate of glucose transport is rather constant, with a $v_{max} = 620\ nmol/min/mg\ protein$, equivalent to $v_{max} = 620 \times 10^{-6} \times 60 \times 500 = 18.6\ mmol/gDW/h$, which is close to the maximal glucose consumption rate of approximately 16 to 19 $mmol/gDW/h$ [49]. With an average $k_{cat} = 125\ s^{-1}$ of the various glucose transporters, taking the flux interval reported by [49] as the upper bound, we compute that glucose transport covers about 7.5 μm^2. We used this number as upper limit:

$$A\ ([HXT]) \leq 7.5\ \mu m^2$$

(51)

This is an empirical proteome constraint, not based on fundamental limits but on regulatory mechanisms. The fact that it is an active constraint in wild-type yeast is confirmed by laboratory evolution experiments in glucose limited chemostats, which have consistently showed increases in high-affinity glucose transporter expression[50].

Proteome Constraint 7: Unspecified protein

The model does not include a complete list of proteins that might be expressed in the organism: only proteins known to catalyze metabolic reactions are included in genome-scale metabolic models. Thus we need to account for the synthesis of proteins required for storage, signaling or maintaining the structure

of the cell. This is especially important at low growth rates, where only low amounts of enzymes are needed to sustain the low metabolic fluxes.

In the biomass reaction, the composition of amino acids is given with stoichiometric coefficients in $mmol/gDW$. We multiplied these coefficients with the molecular weight (MW) of each of the amino acids ($g/mmol$), to convert the amino acid composition r as $mmol\ aa/mmol$ of total amino acids. As a proxy for the proteins we do not explicitly account for, and assuming the length of an average protein to be around 450 amino acids, we designed a dummy protein YDRUMD1. Therefore, the number of residues of a certain amino acid in the unspecified protein YDRUMD1 chain equals to $450 \times r$. Additionally, we set that YDRUMD1 has 50 nucleotides in its 5'UTR, the median for other proteins.

Even in high growth rates, this *unspecified protein* (UP) should be still expressed to a certain degree, as otherwise all the proteome would be occupied by metabolic enzymes – being a rather unrealistic scenario. Using the proteomics data, we estimated about $0.25\ g/g\ Prot$ of proteins. This decision was based on the comparison of proteome fractions, allocated to different cellular processes ($g/g\ total\ protein$), from experimental data and model predictions (Supplementary Figure 1).

For this, we attributed proteins to coarse proteome sectors. The first category of proteins was associated with proteome turnover (similar to Terry Hwa's "R" fraction): translation factors, ribosomal proteins, chaperones, proteasome subunits/proteases (in all cases, including not only cytoplasmic, but also their mitochondrial counterparts). The remaining proteins from experimental datasets were attributed to another category ("Rest of cellular processes", a combination of Hwa's "P" and "Q" sectors). In the case of experimental data (points in the Supplementary Figure 1), these proteins comprised not only metabolic proteins (shown as orange triangles in the plot) but also proteins with other functions (such as signaling and structural proteins).

Then we compared the experimental measurements with model simulations, where the lines show either, again, the proteome fraction of proteins, involved in protein turnover, or - for the "Rest of cellular processes" - the sum of proteome fractions occupied by metabolic proteins and the UP. Notably, with the minimal UP value of $0.25\ g\ UP/g\ protein$, we do see an agreement of the model predictions to the experimental data for both the protein fractions, assigned to both "Proteome turnover" and "Rest of cellular processes" categories. Therefore, we believe that the data supports our decision to use the UP value of $0.25\ g\ UP/g\ protein$ for model simulations.

Thus, we set the minimal requirement of the UP in the proteome to $0.25\ g/g\ Prot$:

$$\frac{v_{syn,UP}}{\mu + k_{deg}}[mmol/gDW] \times \frac{MW_U[g/mol]}{1000\ [mmol/mol]} \geq UP\ [g/g\ prot]\ \times\ prot\ ratio\ [g\ prot/gDW] \qquad (52)$$

Subsequently, the balance between the degradation and dilution of the *UP* is defined as follows:

$$v_{deg,U} = k_{deg,U}\frac{v_{syn,U}}{\mu+k_{deg,U}} \text{ and } v_{dil,U} = \mu\frac{v_{syn,U}}{\mu+k_{deg,U}}$$

(53)

Proteome Constraint 8: Gratuitous protein expression

We included descriptions of some gratuitous proteins (GFP, YFP, mCherry and haemoglobin) into the model to simulate stress response to gratuitous protein expression. As for other proteins, we added protein translation, folding, degradation and dilution-by-growth reactions. The fraction of the total proteome that the gratuitous protein occupies is described by the following constraint (taking $mCherry$ as the example):

$$\frac{v_{syn,mCherry}}{\mu + k_{deg}} \left[\frac{mmol}{gDW}\right] \times \frac{MW_{mCherry} \left[\frac{g}{mol}\right]}{1000 \left[\frac{mmol}{mol}\right]} = mCherry \left[\frac{g}{g\ prot}\right] \times prot\ ratio \left[\frac{g\ prot}{gDW}\right]$$

(54)

Similarly to the UP, the balance between the degradation and dilution of the $mCherry$ is defined as follows:

$$v_{deg,mCherry} = k_{deg,mCherry} \frac{v_{syn,mCherry}}{\mu + k_{deg,mCherry}} \text{ and } v_{dil,mCherry} = \mu \frac{v_{syn,mCherry}}{\mu + k_{deg,mCherry}}$$

(55)

Inhibition of growth (reduction in maximal achievable growth rate) by gratuitous protein expression is simulated by pre-setting the fraction of proteome which gratuitous protein occupies, and running the binary search in order to find the highest growth rate, still resulting in a feasible linear program (just as for unperturbed state).

2.5 Growth-related constraints

Growth-related Constraint 1: Biomass equation

The biomass reaction describes the amount of biomass precursors as $mmol/gDW$ [51]. [52] described how the concentrations these precursors were computed from published data. The composition of carbohydrates, proteins (as amino acids), DNA, and RNA (as [deoxy]ribonucelotides) is included to the biomass equation.

In the biomass equations of the conventional GEMs, the stoichiometric coefficients of the precursors do not change with the growth rate. However, the biomass composition is known to vary from strain to strain and, in the case of CEN.PK113-7D, was observed to be a function of growth rate[40]. Thus, we used the data of [40] to obtain the regression curves for the growth rate-dependent change in bulk biomass composition, when the growth rate is determined by different glucose availability (Supplementary Table 1). Thus, for simulating glucose-limited growth, at every simulation step, we set the flux through the new biomass

reaction to be equal to the corresponding growth rate and recompute its composition according to the relationships listed in Supplementary Table 1.

However, it is known that cells, treated with the translation inhibitor cycloheximide do not accumulate storage carbohydrates despite the halted growth[53]. Thus for simulating inhibition of ribosomes, we set the biomass composition to be that of $\mu = 0.40\ h^{-1}$ at all times. Notably, we also performed analogous ribosome inhibition simulations where the biomass composition was changing depending on the growth rate μ as described above, however, that did not have a major effect to the outcome of the simulations.

Supplementary Table 1. Dependency of the abundance of biomass precursors on the growth rate changed by glucose availability.

Component	Regression equation (or fixed amount) $f = [g/gDW]$
Protein	$f_{p,biomass} = 0.4854\,\mu + 0.3888$
Lipids	$f_{lipid} = -0.0456\,\mu + 0.0745$
RNA	$f_{RNA} = 0.1525 \times \mu + 0.0498$
Adenylate*	$f_{Ade} = 0.2 \times f_{RNA}$
Guanylate*	$f_{Gua} = 0.283 \times f_{RNA}$
Cytidylate*	$f_{Cyt} = 0.26 \times f_{RNA}$
Uridylate*	$f_{Ura} = 0.20 \times f_{RNA}$
DNA**	$f_{DNA} = 0.005$
Inorganic phosphate	$f_{P_i} = 0.006$
Inorganic sulphate	$f_{SO_4^{2-}} = 0.003$
Carbohydrates (total)***	$f_{carbs} = -0.5249 \times \mu + 0.449$
Trehalose****	$f_{treh} = (-0.555 \times \mu + 0.183) \times f_{carbs}$
Glycogen****	$f_{glyc} = (-0.837 \times \mu + 0.317) \times f_{carbs}$
Mannans	$f_{treh} = (0.511 \times \mu + 0.219) \times f_{carbs}$
Glucans	$f_{treh} = (0.880 \times \mu + 0.281) \times f_{carbs}$

* - data from [54];
** - exact composition of the deoxyribonucleotides taken from the Yeast7.6;
*** - data from [55];
**** - if the computed fraction is less than zero, the value becomes zero;

To define the growth rate-dependent lipid composition, we made a lumped reaction consisting of 64 lipid species. We used the data of [56], where the data were provided for every lipid species as mol/mol lipid. First, we recomputed the molar fractions into mass fractions, using the molecular masses of individual lipids from the Yeast Metabolome Database (YMDB)[57] and LIPID MAPS (http://www.lipidmaps.org/). Then, using the bulk measurements of [40], we distributed the total lipid mass fraction to the individual lipids, including them into the biomass equation in $[mmol/gDW]$.

In the case of inhibition of translation at glucose excess, we kept the biomass composition that corresponds to the uninhibited state.

Growth-related Constraint 2: ATP maintenance

For the growth-related processes that we do not account for explicitly in the model, we need to define an ATP hydrolysis reactions, that are split into a growth-associated ATP maintenance, GAM, and a non-growth associated maintenance reaction NGAM:

$$GAM: ATP + H_2O \rightarrow ADP + P_i + H^+ \tag{56}$$

$$NGAM: ATP + H_2O \rightarrow ADP + P_i + H^+ \tag{57}$$

The GAM value is multiplied by the growth rate ($v_{GAM} \times \mu$), whereas NGAM is fixed to the NGAM value v_{NGAM}. For galactose and maltose growth, the added a third ATP maintenance term cNGAM to test if additional ATP cost would improve the predictions (see Section 5 of this document).

In conventional genome-scale metabolic models, e.g. Yeast7.6, the flux of GAM v_{GAM} at the growth rate μ^* is defined as $v_{GAM}(\mu) = 59.3\ mmol/gDW \ \times \mu$. However, our model already describes many of the ATP-investing processes, as ATP is used in protein translation, folding and degradation. As reported by [10], at $\mu = 0.1\ h^{-1}$, the protein biosynthesis and turnover costs are about 23 and 12 $mmol/gDW$, respectively.

Experimentally, the value of GAM is usually estimated using flux data from glucose-limited chemostats [58,59], and, in the case of aerobic growth, always from fully respiratory data below the critical dilution rate. Following these procedures, we found that a GAM value of $v_{GAM}^{max}(\mu) = 24.0\ mmol/gDW \times \mu$ was able to describe full respiratory growth on glucose very well (Fig. 2 in the main text). However, our initial simulations showed that this value does not fit the data at high growth rates very well: fixing the GAM value resulted in elevated demand of carbon source (in this case, glucose), as well as ca. 30% higher ethanol flux at $\mu \rightarrow 0.4\ h^{-1}$ than experimentally reported (both including our own data and [60]). Thus we concluded, based on the available data, that GAM should be lower at higher growth rates to explain the growth-rate associated fluxes. We have no mechanistic explanation, but since the deviation was the strongest at the highest growth rates, we suspect it to be linked to glucose-dependent signaling. We therefore coupled the GAM value to the saturation level of the hexose transporters f_{sat}^{HXT}:

$$v_{GAM}(\mu, f_{sat}^{HXT}) = v_{GAM}^{max} \times \mu \times (1 - 0.5 f_{sat}^{HXT}) \tag{58}$$

As the saturation of the hexose transporters f_{sat}^{HXT} is low, the $v_{GAM} \approx 24.0\ mmol/gDW \times \mu$; low saturation rates ($f_{sat}^{HXT} < 0.20$) indeed correspond to the glucose-limited growth with $D < D_{crit}$, keeping the consistency with the experimental observations. As $f_{sat}^{HXT} \rightarrow 1$, $v_{GAM} \rightarrow 0.5\ v_{GAM}^{max} \times \mu$, or $v_{GAM} \approx 12\ mmol/gDW \times \mu$.

To estimate the value of the NGAM, we used the flux data from glucose-limited conditions: aerobic cultivation in chemostat[40,60] and this study, as well as cultivation in anaerobic chemostats[61]. Plotting the $v_{ATP}(\mu)$, we found an NGAM value of $v_{ATP}(0) = v_{NGAM} = 0.818\, mmol/gDW/h$ (Supplementary Figure 13). However, this ATP expenditure still included the costs of synthesis and degradation of the proteins at near-zero growth. To account to this, we computed the respective requirements of ATP ($v_{ATP}^{p-syn}(0) = 0.486\, mmol/gDW/h$ and $v_{ATP}^{p-deg}(0) = 0.164\, mmol/gDW/h$) and, by subtracting these components from the total NGAM, we set the $v_{NGAM} = 0.168\, mmol/gDW/h$ in our model.

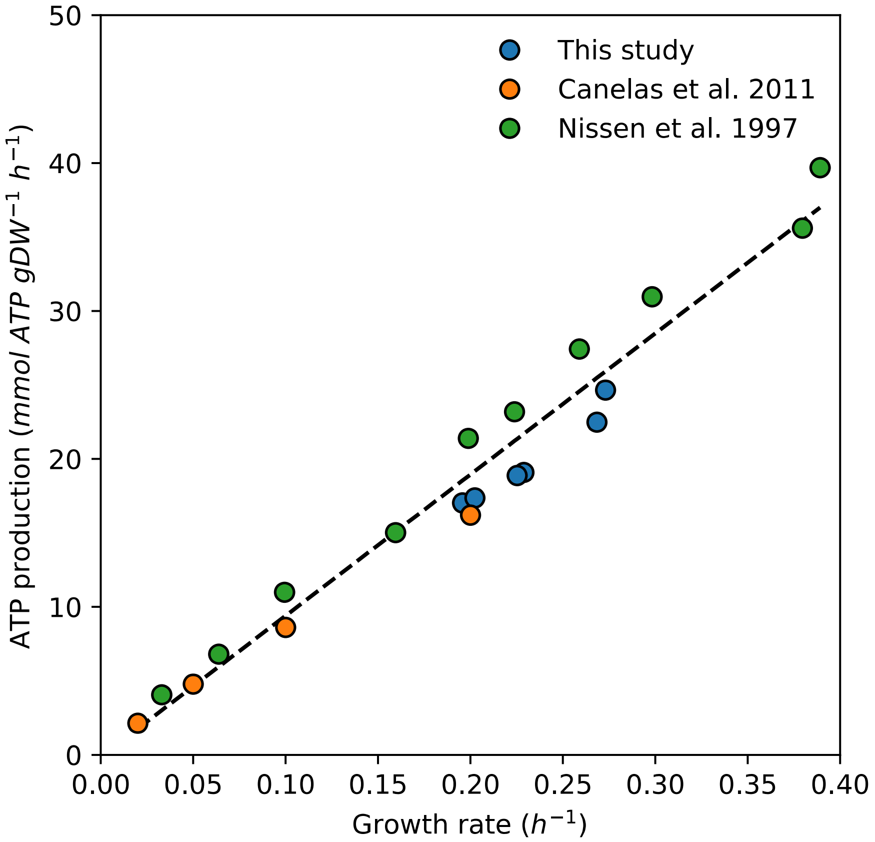

Supplementary Figure 13. Computed ATP production for different growth rates in anaerobic[61] and aerobic ([40], this study) settings. ATP production was computed as $v_{ATP}^{aerobic} = 2 \times v_{Glc} + \frac{1}{3} \times v_{CO_2} + 2 \times v_{O_2}$ for aerobic conditions (representing the production by glycolysis, TCA cycle and oxidative phosphorylation, respectively) and $v_{ATP}^{anaerobic} = 2 \times v_{Glc} - v_{Glyc}$ (production by glycolysis, corrected for carbon, rerouted to glycerol).

Growth-related Constraint 3: Growth media composition (exchange reactions)

The minimal growth medium we considered contained glucose, sulphate, ammonia, and phosphate. To mimic different growth media, we changed the upper and lower bounds for exchange reactions of the metabolites, according to the descriptions of the SC and YPD growth media[62]. For the minimum glucose

medium, we set both lower and upper flux bounds to zero for all exchange reactions for amino acids and other metabolites, which are otherwise present in rich growth media. For simulating anaerobic growth, the minimal glucose-based media was also supplemented with trace amounts of ergosta-5,7,22,24(28)-tetraen-3beta-ol (an ergosterol derivative, crucial for growth), palmitoleate and oleate (the latter two are experimentally provided with addition of Tween 80 to the growth medium).

In most of the experiments we performed ourselves and collected from literature sources, glucose was used as the sole (or main, in case of rich SC/YPD media) carbon source. Thus, assessment of residual glucose levels are an important consideration on correctly predicting metabolic behaviour. We computed the concentration of residual glucose from the model simulations, using formula $[Glc_{Res}] = \frac{f_{sat}}{1-f_{sat}} \times K_M$, with f_{sat} being the saturation factor of the glucose transporters and $K_M = 1\,mM$ being the Michaelis constant of high-affinity glucose transporters. Predictions of the residual glucose levels agree to the experimental observations that suggest only minute amounts of glucose in spent media from glucose-limited chemostats (see Fig. 2a of the main text).

We modeled glucose uptake as irreversible process and, as a result, obtained good agreement with the experimental data on both the residual glucose concentrations and glucose uptake rates. Therefore, we conclude that we can safely neglect the potential inhibitory effects of intracellular glucose concentration. In glucose-limited chemostats, the concentration of intracellular glucose is expected to be very low and the effect therefore very small. It should be noted that a strong impact of intracellular glucose would only complicate the mapping between saturation level of the transporter and the extracellular glucose concentration, but not the effect of transport saturation on growth rate and metabolic strategies.

Growth-related Constraint 4: Energetics of oxidative phosphorylation

Measurements of ATP yields from oxidative phosphorylation in S. cerevisiae show that this process is far away of its theoretical yield of ca. $32\,ATP/Glc\ [mol/mol]$. Rather than that, usually reported value is twice as small, ca. $16\,ATP/Glc$ [63]. However, in the original Yeast7.6 model, maximal yield of ATP is $22\,mol\,ATP/mol\,Glc$ ($\max ATPase$, $v_{Glc} = 1\,mmol/gDW/h$, exchange for O_2, and inorganic ions unbound).

[63] argued that one of the main factors leading to low yield of ATP from oxidative phosphorylation is proton leaks through the inner mitochondrial membrane from the intermembrane space towards the matrix. This phenomenon was observed already decades ago[64], was shown to be dependent on the intensity of respiration[65] and was suggested to be an active process[66].

To account for this process, we implemented a linear constraint, which forces two protons to leak from intermembrane space to the mitochondrial matrix for every pair of electrons transferred from NADH onto ubiqui-6-one. In Yeast7.6, such constraint changes the ATP yield to $16\,mol\,ATP/mol\,Glc$.

Supplementary Note 3. Manual curation of turnover values

3.1 Maltose-specific metabolism

Compared to glucose, to shuttle maltose into glycolysis, two new enzymes have to be expressed: a maltose-specific transporter MAL11 and maltase MAL12. Even though we collected a high number of turnover values, there is still high uncertainty about many of these enzyme turnover values, which are directly linked to the protein costs in our model. We noticed inconsistencies between the retrieved turnover values and experimental data for maltase: based on experimental data, in maltose-grown cells, enzyme maltase makes up about 2% of the total proteome. Meanwhile, the reported turnover value was $k_{cat}^{GECKO} = 1278.0\ \mathrm{s}^{-1}$, which is unrealistically high given the measured proteome fraction. Therefore, we manually adjusted the turnover values of the maltase using our proteomics data.

We first obtained values for v_{max} for individual enzymes from reports in the literature. We then computed the amount of enzyme in the dry mass of *S. cerevisiae* $[e_i]$ from our proteomics data, using the relationship between the growth rate and protein content in dry mass from [40] as follows: by knowing the proteome fraction in $g/g\ protein$ and the total amount of proteins in dry biomass ($g\ protein/gDW$), we computed the mass of protein of interest m_i (in g/gDW), which we converted into amount of protein $[e_i]$ (in $mmol/gDW$) using its molecular weight. Assuming that the enzymes of interest are bottlenecks in their respective pathways and thus are fully saturated, we followed the relationship $v_{max} = k_{app} \times [e_i]$, and computed the k_{app} values using the maximal flux values v_{max} we collected (Supplementary Table 2).

We also compared the expression levels and measured turnover values for galactose-specific proteins (the Leloir pathway). Yet, we did not notice striking differences and thus did not adjust the turnover values.

Supplementary Table 2. Estimated k_{eff} value for maltase.

Gene	Protein	Functionality	Proteome mass fraction	k_{cat} (GECKO) (s^{-1})	k_{eff} (s^{-1})	k_{cat} $/k_{eff}$	Source
MAL12	YGR292W	Maltase	0.0201	1278.0	2.258	565.99	doi

3.2 Other metabolic enzymes

For collection of the k_{cat} values for metabolic enzymes, we used the GECKO toolbox[11]. For the proteins without a GECKO-provided k_{cat} value, we assumed a default of $k_{cat} = 90\ s^{-1}$. However, during the process of automatic compilation of the turnover values, some non-physiological measurements are acquired (e.g. turnover number of a mutant protein, or a protein with the same function from a very distant species). Thus we also performed manual curation of the k_{cat} values for the following enzymes (Supplementary Tables 3 and 4).

Supplementary Table 3. Manually curated turnover values (k_{cat} [cur.]) for metabolic enzymes.

Gene	Protein	Functionality	k_{cat} (GECKO) (s^{-1})	k_{cat} (cur.) (s^{-1})	Source
PMA1	YGL008C	H^{+}-ATPase	56.8	36.0	doi
		ATP synthase	20.0	120.0	doi
GLT1	YDL171C	Glutamate synthase	70.0	52.7	doi
GLN1	YPR035W	Glutamine synthase	52.7	7.02	
GDH3<br>*GDH1*	YAL062W<br>YOR375C	$NADP^{+}$-dependent GDH	2682	13.0	doi
AQY2	YLL052C	Aquaporin	90.0	900.0	Unrealistic model predictions (futile cycle)
DLD2<br>*CYC7*	YDL178W<br>YEL039C	D-lactate dehydrogenase	1500.0	81.0	Unrealistic model predictions (artificial pathway)
GCV3<br>*GSD1*<br>*LPD1*<br>*GSD2*	YAL044C<br>YDR019C<br>YFL018C<br>YMR189W	Glycine cleavage system	1500.0	1.0	Unrealistic model predictions (futile cycle)
SMH1	YBR263W	Serine hydroxymethyltransferase	640.0	2.0	doi

Supplementary Table 4. Manually curated plasma membrane transporters.

Metabolite	Comment	kcat (s^{-1})
Glucose	There are many different glucose transporters with different k_{cat} and K_M values. [67] and [68] estimated that the turnover constants for glucose transporters HXT7 and HXT2 are equal to 200 and 53 s^{-1}, respectively. Rather than specifying the K_M values, we used a varying saturation level to change glucose availability in simulations, and used an average k_{cat} of glucose transporters $k_{cat,Glc} = 125\ s^{-1}$.	125
Phosphate	[69] reported that the phosphate transporter Pho84 has the specific activity as 6 $\mu mol/gDW/min$. The concentration of Pho84 is about 300,000 molecules/cell [70], so we can estimate the k_{cat} of Pho84 as:	3

	$$k_{cat} = \frac{\frac{6.1\ mmol}{1000\ gDW\ min}}{\frac{300{,}000}{13 * 6.023 \times 10^{8}}\ \frac{mmol}{gDW}} = 159.2\ min^{-1} \approx 3\ s^{-1}$$	
Ammonium	[71] measured the rate of transported ammonium as 30 to 300 ions per second.	30
Sulphate	Default value for proteins without GECKO-provided turnover number	90

Supplementary Note 4. Identification of active Elementary Flux Modes

In the Main Text (see "Changes in metabolic strategies are the result of proteome constraints"), we analyzed the model to understand why the metabolic fluxes changed with glucose availability. For this we made use of earlier developed mathematical theory about the solutions of growth rate maximization that use rates as optimization variables and weighted sums of proteins as constraints[72,73]. Optimal solutions of such maximization problems are superpositions of so-called Elementary Growth Modes (EGMs), whose metabolic subnetwork can be described by long-known Elementary Flux Modes (EFMs) under constant amino acid composition of the biomass[74].

EFMs can be seen as metabolic pathways with fixed stoichiometry between its substrates and products, and they are elementary in the sense that each reaction in an EFM is essential to maintain steady state[73]. It was mathematically proven that the number of EFMs that can carry flux in optimal solutions is bounded by the number of constraints that actively limit the maximal growth rate[72]. Which specific flux modes, and hence metabolic strategies, are optimal, depends on the cost-effectiveness (or "proteome efficiency" [2,75]) of each mode.

The detailed mapping of all processes in protein synthesis and degradation allows pcYeast to compute the costs of implementing these strategies. In this section, we present the procedure to identify the number of active Elementary Flux Modes (EFMs) in (proteome-constrained) genome-scale models, stressing particularities of this procedure that are specific for the pcYeast model.

As a side note, we first have to discriminate between two types of constraints that we apply to the model: equality constraints and *in*equality constraints. Equality constraints have to be always met for any optimization, while inequality constraints can obtain any values bound by both flux definitions and the right-hand-side. For an inequality constraint on fluxes v with scaling factors w: $\sum w_i v_i \leq V$, with all $v_i \geq 0$ and $V \geq 0$, the value of the left-hand-side post-optimization can obtain values in the range of $[0; V]$. We call such an inequality constraint "active" if $\sum w_i v_i = V$ post-optimization.

Following the method initially presented by [76], we begin the enumeration of active EFMs with optimizing the linear program which results in a flux distribution with all non-zero flux values. It should be noted here that all fluxes should be non-negative $v_i \geq 0$ (i.e. reversible reactions are split into two irreversible reactions) and in the pcYeast model, all reversible reactions are split into two unidirectional reactions already (see **Mass Balance Constraint 1**). Note that this list of non-zero fluxes, which we call "active fluxes" will contain either metabolic fluxes alone (conventional genome-scale models) or both metabolic and proteome-related fluxes (in the case of pcYeast). Here we aim to enumerate EFMs and not EGMs[74], and therefore we will consider only the active metabolic fluxes in pcYeast.

We first need to obtain the stoichiometric matrix; we can use the model handling tools, like PySCeS CBMPy[8], to generate the stoichiometric matrix of the model and export it to a text file, which can be handled independently of the software used to run models. Note that for the pcYeast model, we first need to compute the growth rate-dependent stoichiometric coefficients for biomass equation and add the reaction itself, only then export the matrix. The resulting matrix will have a shape of m rows, representing metabolites, and n columns, representing reactions. Having both the list of active metabolic fluxes and

the stoichiometric matrix, we can subset the stoichiometric matrix to include only the active fluxes, which we call "active stoichiometric matrix".

We can compute the number of active EFMs by computing the rank of the active stoichiometric matrix or by enumerating EFMs using software packages, like *efmtool*[77]. By using the rank computation approach, we compute the rank of the active stoichiometric matrix. The difference between the rank (the number of independent mass balances, i.e. equations) and the number of reactions (the variables) determines the degrees of freedom. Since EFMs have by definition only one degree of freedom, and every flux distribution is a linear combination of EFMs[76], the number of degrees of freedom must equal the number of active EFMs. We have previously reported that such an approach works for conventional genome-scale models of yeast that do not contain equality constraints[78].

Applying the rank computation routine is not entirely trivial for the pcYeast model. We performed a computation described above for a glucose-limited condition at low growth rate, where we were expecting a single EFM to be active, based on the observations of number of active compartment-specific proteome constraints, together with stable biomass yields of $Y_{X/S} \approx 0.52\ g/g\ Glc$ and exchange fluxes scaling uniformly with the growth rate (see Fig. 2 in the Main Text). We obtained the difference between the number of active fluxes and the rank of $n - 6$, suggesting 6 EFMs to be active. This result was puzzling at first, but we can show that our claim for the number of growth-supporting EFMs equaling 1 is in fact correct, by taking into account the equality constraints.

In pcYeast we set a number of equality constraints, which represent 3 types of ATP maintenance ($NGAM$, GAM, mitochondrial maintenance) and another constraint, representing the proton leak in the mitochondrial inner membrane (see **Growth-related Constraint 3** and **4**, **Proteome Maintenance Constraint 3**). Since the fluxes through the corresponding reactions are either determined prior to optimization (when constraint expression is $v_1 = value$) or linked to other fluxes in the system (e.g. $v_1 = value \times v_2 > 0$), these fluxes are either known both pre- and post-optimization, or can be computed post-optimization from other fluxes. Either way, these fluxes provide additional degrees of freedom in the rank computation.

We have removed each of the equality constraints described above one-by-one, and at each step, the difference between the number of active fluxes and the rank of the corresponding active stoichiometric matrix would be lower by 1. With this, we validated that every additional equality constraint introduces a degree of freedom: with all 4 constraints removed, the rank of the active stoichiometric matrix was $(n - 2)$. The final additional degree of freedom comes from the fact that we fix the value of the biomass flux to the growth rate we simulate at every iteration (**Growth-related Constraint 1**). However, to maintain the steady-state nature of the system, we naturally cannot remove this flux to arrive to the difference of $(n - 1)$.

With some modifications specific to the pcYeast structure we could verify that the result of $(n - 2)$ does correspond to a single EFM, using an alternative approach with the *efmtool*. By running the *efmtool* on the active stoichiometric matrix, the user receives not only the number of EFMs, but also the flux values for every non-zero flux belonging to each of the EFMs. For *efmtool*, we considered that the flux distributions we compute include costs of protein production, both in terms of amino acids and energy. It is important to augment the biomass equation with charged tRNAs as substrates and free tRNAs as products. Moreover, while this is accounted for in the cytoplasmic part of the model, for the sake of

completeness, hydrolysis of (even a small amount of) mitochondrial GTP must be included into the biomass equation as well. Subjecting this modified active stoichiometric matrix to the *efmtool* results in detection of one active EFM, containing all the active fluxes with non-zero values.

Thus, by either rank computations or analysis by *efmtool*, we indeed can show that with one compartment-specific proteome constraint active, the predicted flux distribution can be described as a single EFM. The procedure works also well for situations where other proteome constraints are active: for instance, in the case of growth at glucose-excess, the pcYeast suggests only one compartment-specific proteome constraint, minimal UP fraction, to be active. Enumeration of EFMs suggested a single EFM being present, in line with the previously provided example of glucose transporter-limited growth.

Supplementary Note 5. Growth on galactose and maltose

We aimed to simulate batch growth of *S. cerevisiae* on different other hexose sugars, at first with the model that we calibrated with glucose chemostat data without any adjustments ("naïve"), and using the original GECKO-based turnover values for the metabolism of galactose and maltose (except for maltase, see Section 3.1). However, the flux distributions and the growth rates we obtained largely resembled these of glucose excess conditions (Supplementary Table 5), suggesting that the naïve model cannot capture the effects of growth on sugars other than glucose.

Supplementary Table 5. Comparison of the experimental measurements and predicted fluxes by the naïve models for growth in excess sugar conditions. Fluxes are in $mmol/gDW/h$ except for the flux through the biomass reaction (the specific growth rate, in h^{-1}).

Substrate	Flux	Experimental data	Naïve prediction
Glucose	$\mu\ (h^{-1})$	0.390	0.400
	qS_x	13.5	13.82
	q_{O_2}	2.0	3.23
	q_{EtOH}	19.0	20.90
	q_{CO_2}	22.2	24.78
Maltose	$\mu\ (h^{-1})$	0.283	0.340
	qS_x	5.2	7.90
	q_{O_2}	2.7	2.62
	q_{EtOH}	14.1	25.96
	q_{CO_2}	17.0	29.15
Galactose	$\mu\ (h^{-1})$	0.174	0.350
	qS_x	2.6	7.61
	q_{O_2}	5.3	5.19
	q_{EtOH}	0.7	8.44
	q_{CO_2}	6.0	14.13

We therefore looked for the determinants that could explain the experimentally determined phenotypes of *S. cerevisiae*. First consideration of ours was the maximal uptake of the carbon source: the maximal uptake flux in the naïve model was almost 3-fold and 1.5-fold higher than experimental values for galactose and maltose, respectively. Moreover, the predicted abundance of the galactose- and maltose-specific transporters GAL2 and MAL11, respectively was also between 3-to-10 times higher, compared to quantitative proteomics measurements. Based on these observations, we scanned the maximal area, which hexose transporters can occupy (**Proteome Constraint 6**) (Supplementary Figure 14) in order to estimate a suitable maximal expression level. Following the results of these simulations, we reduced the

maximal area allowed for hexose transporters accordingly: for the galactose case from 7.5 to 3 $\mu m^2/cell$, and for the maltose case from 7.5 to 3.5 $\mu m^2/cell$. It should be noted that we explicitly picked the values which would result in lower uptake fluxes than the experimentally measured.

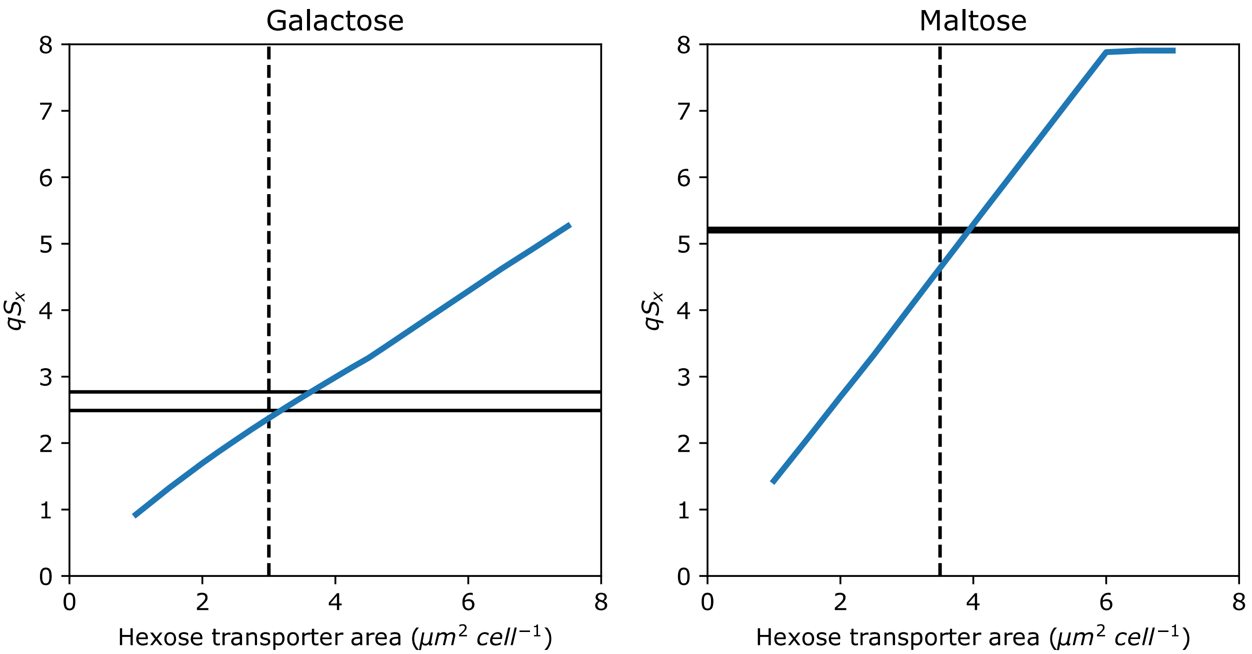

Supplementary Figure 14. Estimation of the maximal area for hexose transporter to occupy. The naïve model was used in simulations with the surface area varying from 1 to 7.5 $\mu m^2/cell$. Solid horizontal lines represent experimentally determined uptake fluxes from individual batch cultures, and dashed vertical lines represent the maximal area which was selected for further experiments.

As we saw a strong negative impact on the experimentally measured growth rate for growth on carbon sources other than glucose, we then postulated that there must be additional effects alongside the apparent expression limit of hexose transporters. Therefore we considered probing how applying additional burden on the total proteome changes the model predictions. This choice was backed by our modeling insight that the growth of *S. cerevisiae* in excess of glucose is limited only by total proteome limitation. For simulations, we used the model and varied the value of minimal unspecified protein (UP) fraction in the proteome in order to impose an additional proteome burden.

Accounting to the initial distance of the naïve predictions from the experimental measurements, we selected to scan different ranges of UP minimal values, between 0.42 and 0.615 $g\ UP/g\ protein$ for galactose. For maltose, we took the same approach but sampled UP minimum between 0.225 and 0.42 $g\ UP/g\ protein$ instead, due to lower reduction of the growth rate, compared to the naïve model. For each of the minimal UP values tested, we acquired the distribution with the highest feasible growth rate. As a criterion of the goodness-of-fit, we then computed the sum of scaled square residuals (compared to the experimental data) based on the flux through the biomass reaction (experimentally determined

growth rate), and exchange fluxes of the carbon source, oxygen, ethanol and carbon dioxide: $RSS = \sum\left(\frac{v_i^{exp} - v_i^{pred}}{v_i^{exp}}\right)^2$ (Supplementary Figure 15), meaning that the lower the RSS value, the closer the prediction is to the experimental measurements.

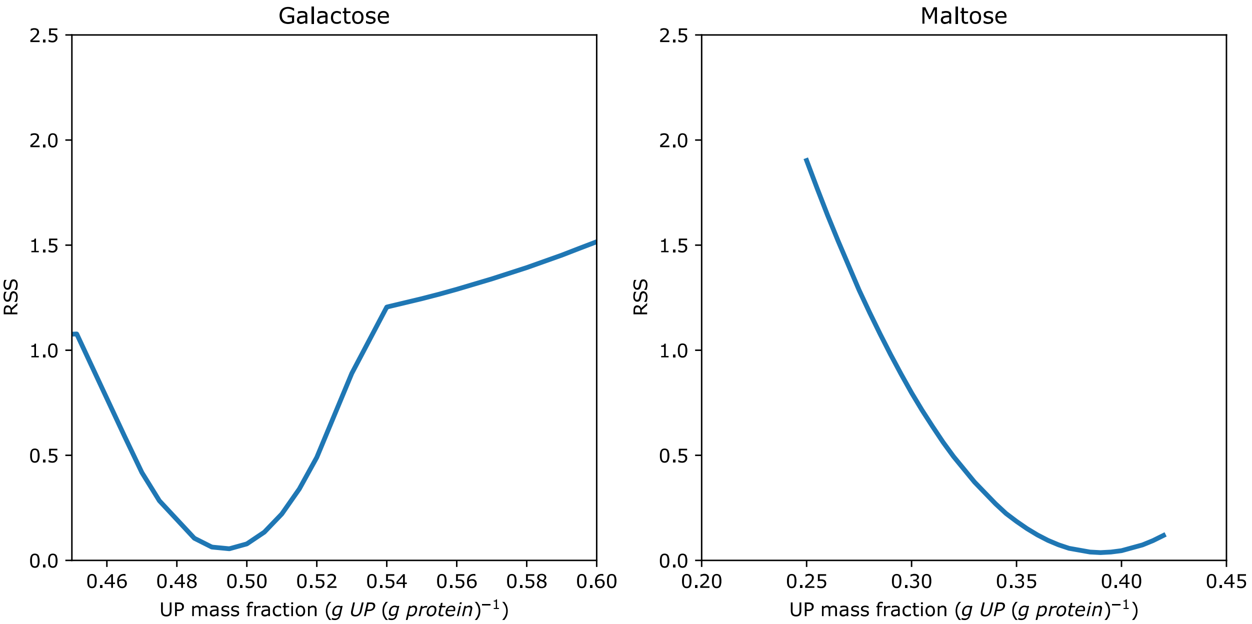

Supplementary Figure 15. Estimation of the minimal UP mass fraction for galactose- or maltose-excess conditions.

For maltose, the goodness-of-fit measure reached values very close to zero, suggesting the flux profile well resembling that of experimental measurements. However, the fit was worse for the galactose case, even with a presence of a clear minimum of the goodness-of-fit measure in the sampled range of minimal UP values. In an attempt to better match the experimental data for galactose, we compared the fluxes of carbon source and oxygen uptake at galactose-excess conditions to glucose-limited chemostat at $D = 0.20\ h^{-1}$. The fact that consumption of the carbon source, as well as oxygen uptake on galactose was higher compared to a glucose-limited chemostat setting with comparable growth rate, suggested an additional energetic cost, which is associated with metabolizing galactose.

To simulate such an effect, we added an extra ATP maintenance reaction to the model, a “carbon-related NGAM” (cNGAM) and performed a parameter scan, similar to a previously described one. For the sake of completeness, we did an analogous scan for maltose as well. We picked cNGAM values from 0 to 5 $mmol/gDW/h$ for galactose and from 0 to 2.5 $mmol/gDW/h$ for maltose, and plotted some of the scan results in Supplementary Figure 16.

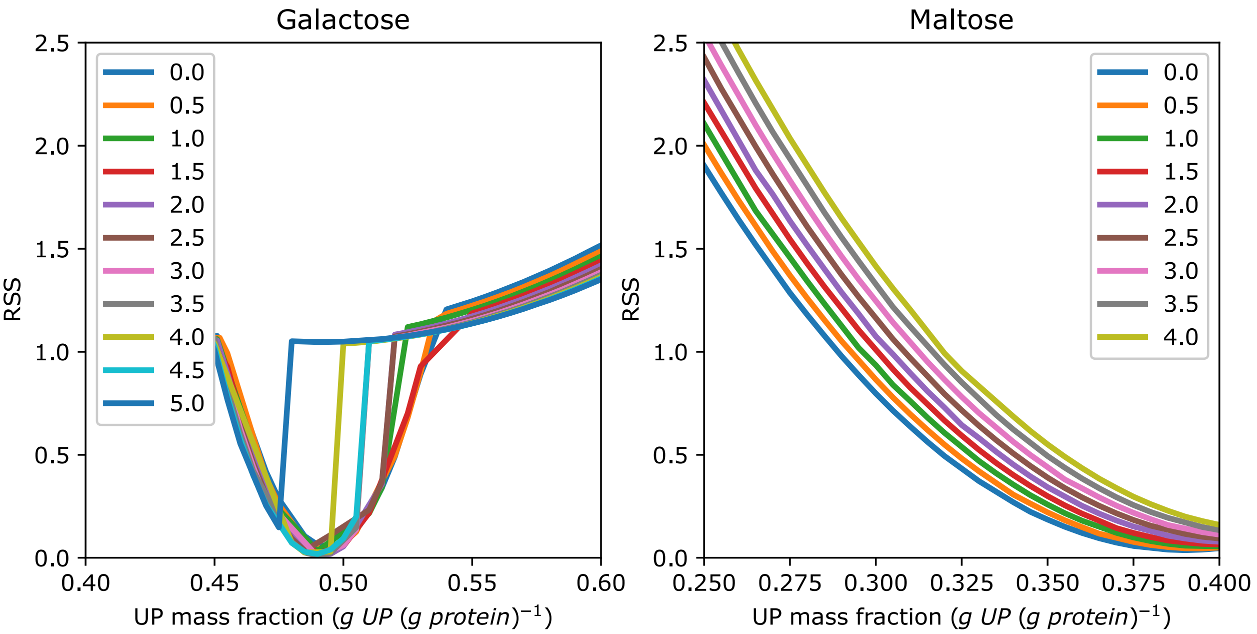

Supplementary Figure 16. Goodness-of-fit for simulations with varying cNGAM value for galactose- and maltose-excess conditions at different UP minima.

For galactose simulations with additional energetic costs added (Supplementary Figure 16, left panel), we observed a further decrease in the RSS values, suggesting an even better fit to the experimental data. To make sure this was indeed the case, we compared the predicted fluxes for galactose with the experimental data to determine the best combination of the two parameters (Supplementary Figure 17).

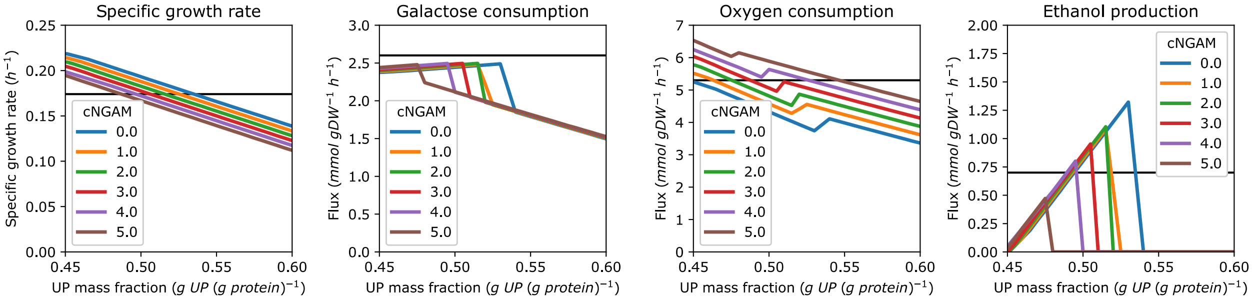

Supplementary Figure 17. The flux profiles of growth on galactose with varying $cNGAM$. Lines, simulated values, horizontal black lines, experimentally measured values.

Thus, for galactose, the set of parameters, which resulted in the closest agreement to the experimental measurements (i.e. lowest RSS value), was the following: 0.49 $g\ UP/g\ protein$ and 3.0 $mmol/gDW/h$ of cNGAM. We listed the predicted specific growth rates and the exchange fluxes for galactose in Supplementary Table 6.

For growth on maltose, additional simulations with non-zero cNGAM still suggested that the increase of the minimal UP value alone was enough to explain the experimental data. Thus we considered changing only the minimal UP value, and introduce no additional energetic cost (i.e. no cNGAM) (FigSM3, right panel). As we introduced additional proteome costs (by adjusting turnover values for maltase) alongside changing the minimal UP value, we wanted to pick a value of minimal UP, which was closest to that of the glucose-calibrated model (corresponding to 0.245 $g\ UP/g\ protein$). By setting a threshold of the $RSS < 0.25$, the first value satisfying this threshold was 0.34 $g\ UP/g\ protein$, which we accepted as sufficient (Supplementary Table 6).

We also attempted to probe the growth on maltose with different combinations of three factors (by, e.g., implementing a change in UP minimum, non-zero NGAM but not fitted turnover value for maltase). Yet, the best agreement to the experimental data was seen in the cases where there was no additional energetic cost, some increase in the minimal UP value and the turnover value was fitted.

Thus, by using a combination of the three factors mentioned above, we show that we can describe the patterns of the metabolic fluxes for hexose sugars other than glucose by changing model parameters. We compared the predicted fractions that proteins with metabolic or unspecified function occupy with the experimental measurements, and the fractions were comparable (Supplementary Table 7). For growth on both galactose and maltose, these changes result in two compartment-specific proteome constraints being active: maximal hexose transporter expression constraint and minimal amount of unspecified protein.

Supplementary Table 6. Comparison of the experimental measurements to the predicted fluxes in the final models. Fluxes are in $mmol/gDW/h$ except for the flux through the biomass reaction (the specific growth rate, in h^{-1}).

Substrate	Flux	Experimental data	Prediction after modifications	Substrate	Flux	Experimental data	Prediction after modifications
**Maltose**	$\mu\ (h^{-1})$	0.283	0.280	**Galactose**	$\mu\ (h^{-1})$	0.174	0.182
	qS_x	5.20	4.88		qS_x	2.6	2.47
	q_{O_2}	2.70	4.06		q_{O_2}	5.3	5.26
	q_{EtOH}	14.1	14.26		q_{EtOH}	0.7	0.69
	q_{CO_2}	17.0	18.79		q_{CO_2}	6.0	6.25

Supplementary Table 7. Comparison of proteome fractions, attributed to metabolic and unspecified proteins.

Carbon source	Fraction of metabolic proteins + UP ($g/g\ protein$)	
	Experimental	Predicted with estimated UP
**Glucose**	0.6809	0.6693
**Galactose**	0.7031	0.7893
**Maltose**	0.6802	0.7362

Supplementary Note 6. Evaluating idle ribosome content in steady-state

In this section, we follow [18] to define a growth-rate dependent fraction of inactive ribosomes in pcYeast. [18] used quantitative proteomics to determine the relationship between the specific growth rate μ and ribosomal proteome fraction ϕ_R for *S. cerevisiae*: $\phi_R = 0.35 \times \mu + 0.08$. This suggests that a constant fraction of ribosomes (0.08 g ribosomal proteins per gram total protein) is present at any non-zero growth rate. Our own data also suggests a presence of certain offset of ribosomal proteome fraction (main text, Fig. 1c).

Considering any growth rate, we can decompose the ribosomal fraction into the fraction of active (translating) ribosomes ϕ_R^A and inactive ones ϕ_R^0: $\phi_R = \phi_R^A + \phi_R^0$. Since proteins are translated by active ribosomes only, we can consider the growth rate to be linearly dependent to the ratio of active ribosomes: $\mu = \gamma \times \phi_R^A$ [79], where γ^{-1} is the time required for one ribosome to replicate itself. Substituting the proteome fraction of active ribosomes into the equation for total ribosomal proteome: $\phi_R = \gamma^{-1} \times \mu + \phi_R^0$.

We can estimate γ by considering the maximal peptide elongation rate $k_{elongation} = 10.5 \frac{aa}{s}$ [80] and the total amino acid amount L per ribosome, including assembly factors, necessary for its formation, which are 12369 and 7383, respectively (information used for calculations from [81,82]). Thus, plugging these values into the relationships, we yield $\gamma = \frac{10.5\frac{aa}{s} \times 3600\frac{s}{h}}{19752\ aa} = 1.9137\ h^{-1}$ and $\gamma^{-1} = 0.5225\ h$.

As we consider a steady-state molecular network, the proteome should also include the ribosome amount which *produces* ribosomes. Thus, we decompose $\phi_R^A = \phi_R^{AP} + \phi_R^{AR} \times \phi_R^0$ where $\phi_R^{AP} = \gamma^{-1} \times \mu$ is the proteome fraction of ribosomes, which produce growth-associated proteins, and another fraction $\phi_R^{AR} = \gamma^{-1} \times \phi_R^0$ is allocated towards production of ribosomes. Under zero-growth conditions, $\phi_R^{AP} = 0$, and the total ribosomal fraction reduces to: $\phi_R = \gamma^{-1} \times \phi_R^0 + \phi_R^0 = 0.08$. Solving for ϕ_R^0 yields the steady-state zero-growth proteome fraction of inactive ribosomes of $\phi_R^0 \approx 0.053$. We further used this number to set the growth rate-independent fraction of non-translating ribosomes.

Supplementary References

1. Frick, O. & Wittmann, C. Characterization of the metabolic shift between oxidative and fermentative growth in Saccharomyces cerevisiae by comparative 13C flux analysis. *Microb. Cell Fact.* (2005) doi:10.1186/1475-2859-4-30.

2. Chen, Y. & Nielsen, J. Energy metabolism controls phenotypes by protein efficiency and allocation. *Proc. Natl. Acad. Sci.* **116**, 17592–17597 (2019).

3. Hong, K. K., Vongsangnak, W., Vemuri, G. N. & Nielsen, J. Unravelling evolutionary strategies of yeast for improving galactose utilization through integrated systems level analysis. *Proc. Natl. Acad. Sci. U. S. A.* (2011) doi:10.1073/pnas.1103219108.

4. Kunkel, J., Luo, X. & Capaldi, A. P. Integrated TORC1 and PKA signaling control the temporal activation of glucose-induced gene expression in yeast. *Nat. Commun.* (2019) doi:10.1038/s41467-019-11540-y.

5. Gleixner, A. *et al. The SCIP Optimization Suite 5.0*. (2017).

6. Wang, H. *et al.* RAVEN 2.0: A versatile toolbox for metabolic network reconstruction and a case study on Streptomyces coelicolor. *PLoS Comput. Biol.* (2018) doi:10.1371/journal.pcbi.1006541.

7. Heirendt, L. *et al.* Creation and analysis of biochemical constraint-based models using the COBRA Toolbox v.3.0. *Nat. Protoc.* (2019) doi:10.1038/s41596-018-0098-2.

8. Olivier, B. G., Rohwer, J. M. & Hofmeyr, J. H. S. Modelling cellular systems with PySCeS. *Bioinformatics* (2005) doi:10.1093/bioinformatics/bti046.

9. Pavlov, M. Y. & Ehrenberg, M. Optimal control of gene expression for fast proteome adaptation to environmental change. *Proc. Natl. Acad. Sci.* **110**, 20527–20532 (2013).

10. Lahtvee, P. J. *et al.* Absolute Quantification of Protein and mRNA Abundances Demonstrate Variability in Gene-Specific Translation Efficiency in Yeast. *Cell Syst.* **4**, 495-504.e5 (2017).

11. Sánchez, B. J. *et al.* Improving the phenotype predictions of a yeast genome-scale metabolic model by incorporating enzymatic constraints. *Mol. Syst. Biol.* **13**, 935 (2017).

12. Nilsson, A. & Nielsen, J. Metabolic Trade-offs in Yeast are Caused by F1F0-ATP synthase. *Sci. Rep.* **6**, 1–11 (2016).

13. Placzek, S. *et al.* BRENDA in 2017: New perspectives and new tools in BRENDA. *Nucleic Acids Res.* (2017) doi:10.1093/nar/gkw952.

14. Wittig, U., Rey, M., Weidemann, A., Kania, R. & Müller, W. SABIO-RK: An updated resource for manually curated biochemical reaction kinetics. *Nucleic Acids Res.* **46**, D656–D660 (2018).

15. Bateman, A. *et al.* UniProt: The universal protein knowledgebase. *Nucleic Acids Res.* (2017) doi:10.1093/nar/gkw1099.

16. Caspi, R. *et al.* The MetaCyc database of metabolic pathways and enzymes-a 2019 update. *Nucleic Acids Res.* (2020) doi:10.1093/nar/gkz862.

17. Burley, S. K. *et al.* RCSB Protein Data Bank: Sustaining a living digital data resource that enables

breakthroughs in scientific research and biomedical education. *Protein Sci.* (2018) doi:10.1002/pro.3331.

18. Metzl-Raz, E. *et al.* Principles of cellular resource allocation revealed by condition-dependent proteome profiling. *Elife* (2017) doi:10.7554/eLife.28034.

19. de la Cruz, J., Karbstein, K. & Woolford, J. L. Functions of ribosomal proteins in assembly of eukaryotic ribosomes in vivo. *Annu. Rev. Biochem.* **84**, 93–129 (2015).

20. Hinnebusch, A. G. The scanning mechanism of eukaryotic translation initiation. *Annu. Rev. Biochem.* **83**, 779–812 (2014).

21. Parky, D., Morrisy, A. R., Battenhouse, A. & Iyer, V. R. Simultaneous mapping of transcript ends at single-nucleotide resolution and identification of widespread promoter-associated non-coding RNA governed by TATA elements. *Nucleic Acids Res.* **42**, 3736–3749 (2014).

22. Engel, S. R. *et al.* The Reference Genome Sequence of *Saccharomyces cerevisiae* : Then and Now. *G3: Genes|Genomes|Genetics* **4**, 389–398 (2014).

23. Berthelot, K., Muldoon, M., Rajkowitsch, L., Hughes, J. & McCarthy, J. E. G. Dynamics and processivity of 40S ribosome scanning on mRNA in yeast. *Mol. Microbiol.* **51**, 987–1001 (2004).

24. Dever, T. E. & Green, R. The elongation, termination, and recycling phases of translation in eukaryotes. *Cold Spring Harb. Perspect. Biol.* **4**, 1–16 (2012).

25. Andersen, C. B. F. *et al.* Structure of eEF3 and the mechanism of transfer RNA release from the E-site. *Nature* **443**, 663–668 (2006).

26. Chakraburtty, K. Translational regulation by ABC systems. *Res. Microbiol.* **152**, 391–399 (2001).

27. Shoemaker, C. J. & Green, R. Kinetic analysis reveals the ordered coupling of translation termination and ribosome recycling in yeast. *Proc. Natl. Acad. Sci. U. S. A.* (2011) doi:10.1073/pnas.1113956108.

28. Woolford, John Jr, Baserga, S. J. Ribosome Biogenesis in the Yeast. **195**, 643–681 (2013).

29. Woellhaf, M. W., Hansen, K. G., Garth, C. & Herrmann, J. M. Import of ribosomal proteins into yeast mitochondria. *Biochem. cell Biol. - Biochim. Biol. Cell.* **10**, 1–10 (2014).

30. Greber, B. J. & Ban, N. Structure and Function of the Mitochondrial Ribosome. *Annu. Rev. Biochem.* **85**, 103–32 (2016).

31. Gong, Y. *et al.* An atlas of chaperone-protein interactions in Saccharomyces cerevisiae: implications to protein folding pathways in the cell. *Mol. Syst. Biol.* **5**, 275 (2009).

32. Lopez-Buesa, P., Pfund, C. & Craig, E. A. The biochemical properties of the ATPase activity of a 70-kDa heat shock protein (Hsp70) are governed by the C-terminal domains. *Proc. Natl. Acad. Sci. U. S. A.* (1998) doi:10.1073/pnas.95.26.15253.

33. Rosam, M. *et al.* Bap (Sil1) regulates the molecular chaperone BiP by coupling release of nucleotide and substrate. *Nat. Struct. Mol. Biol.* (2018) doi:10.1038/s41594-017-0012-6.

34. Finley, D., Ulrich, H. D., Sommer, T. & Kaiser, P. The ubiquitin-proteasome system of Saccharomyces cerevisiae. *Genetics* **192**, 319–360 (2012).

35. Peth, A., Nathan, J. A. & Goldberg, A. L. The ATP costs and time required to degrade ubiquitinated proteins by the 26 S proteasome. *J. Biol. Chem.* **288**, 29215–29222 (2013).

36. Patterson-Ward, J., Huang, J. & Lee, I. Detection and characterization of two ATP-dependent conformational changes in proteolytically inactive Escherichia coli lon mutants by stopped flow kinetic techniques. *Biochemistry* (2007) doi:10.1021/bi701649b.

37. Hong, K. K., Hou, J., Shoaie, S., Nielsen, J. & Bordel, S. Dynamic 13C-labeling experiments prove important differences in protein turnover rate between two Saccharomyces cerevisiae strains. *FEMS Yeast Res.* **12**, 741–747 (2012).

38. Fox, T. D. Mitochondrial protein synthesis, import, and assembly. *Genetics* **192**, 1203–1234 (2012).

39. Okamoto, K. *et al.* The protein import motor of mitochondria: A targeted molecular ratchet driving unfolding and translocation. *EMBO J.* (2002) doi:10.1093/emboj/cdf358.

40. Canelas, A. B., Ras, C., ten Pierick, A., van Gulik, W. M. & Heijnen, J. J. An in vivo data-driven framework for classification and quantification of enzyme kinetics and determination of apparent thermodynamic data. *Metab. Eng.* (2011) doi:10.1016/j.ymben.2011.02.005.

41. Erickson, H. P. Size and shape of protein molecules at the nanometer level determined by sedimentation, gel filtration, and electron microscopy. *Biol. Proced. Online* **11**, 32–51 (2009).

42. Tyson, C. B., Lord, P. G. & Wheals, A. E. Dependency of size of Saccharomyces cerevisiae cells on growth rate. *J. Bacteriol.* (1979) doi:10.1128/jb.138.1.92-98.1979.

43. Visser, W. *et al.* Effects of growth conditions on mitochondrial morphology inSaccharomyces cerevisiae. *Antonie Van Leeuwenhoek* **67**, 243–253 (1995).

44. Lynch, M. & Marinov, G. K. Membranes, energetics, and evolution across the prokaryote-eukaryote divide. *Elife* **6**, 1–29 (2017).

45. Uchida, M. *et al.* Quantitative analysis of yeast internal architecture using soft X-ray tomography. *Yeast* (2011) doi:10.1002/yea.1834.

46. Di Bartolomeo, F. *et al.* Absolute yeast mitochondrial proteome quantification reveals trade-off between biosynthesis and energy generation during diauxic shift. *Proc. Natl. Acad. Sci. U. S. A.* (2020) doi:10.1073/pnas.1918216117.

47. Krauss, S. *Mitochondria : Structure and Role in Respiration*. *ENCYCLOPEDIA OF LIFE SCIENCES* (2001).

48. VanKuyk, P. A. *et al.* Aspergillus niger mstA encodes a high-affinity sugar/H+ symporter which is regulated in response to extracellular pH. *Biochem. J.* (2004) doi:10.1042/BJ20030624.

49. Blank, L. M. & Sauer, U. TCA cycle activity in Saccharomyces cerevisiae is a function of the environmentally determined specific growth and glucose uptake rates. *Microbiology* (2004) doi:10.1099/mic.0.26845-0.

50. Ferea, T. L., Botstein, D., Brown, P. O. & Rosenzweig, R. F. Systematic changes in gene expression patterns following adaptive evolution in yeast. *Proc. Natl. Acad. Sci. U. S. A.* (1999) doi:10.1073/pnas.96.17.9721.

51. Thiele, I. & Palsson, B. Ø. A protocol for generating a high-quality genome-scale metabolic reconstruction. *Nat. Protoc.* **5**, 93–121 (2010).

52. Forster, J., Famili, I., Palsson, B. O. & Nielsen, J. Genome-Scale Reconstruction of the Saccharomyces Cerevisie Metabolic Network. *Genome Res.* 244–253 (2003) doi:10.1101/gr.234503.complex.

53. Thomsson, E., Svensson, M. & Larsson, C. Rapamycin pre-treatment preserves viability, ATP level and catabolic capacity during carbon starvation of Saccharomyces cerevisiae. *Yeast* (2005) doi:10.1002/yea.1219.

54. Tada, M. & Tada, M. The base composition of the fractionated soluble ribonucleic acids of yeast. *Journal of Biochemistry* (1962) doi:10.1093/oxfordjournals.jbchem.a127505.

55. Lange, H. C. & Heijnen, J. J. Statistical reconciliation of the elemental and molecular biomass composition of Saccharomyces cerevisiae. *Biotechnol. Bioeng.* (2001) doi:10.1002/bit.10054.

56. Ejsing, C. S. *et al.* Global analysis of the yeast lipidome by quantitative shotgun mass spectrometry. *Proc. Natl. Acad. Sci. {U.S.A.}* **106**, 2136–2141 (2009).

57. Ramirez-Gaona, M. *et al.* YMDB 2.0: a significantly expanded version of the yeast metabolome database. *Nucleic Acids Res.* **45**, gkw1058 (2016).

58. Verduyn, C., Postma, E., Scheffers, W. A. & Van Dijken, J. P. Physiology of Saccharomyces cerevisiae in anaerobic glucose-limited chemostat cultures. *J. Gen. Microbiol.* (1990) doi:10.1099/00221287-136-3-395.

59. Verduyn, C., Postma, E., Scheffers, W. A. & Van Dijken, J. P. Physiology of Saccharomyces cerevisiae in anaerobic glucose-limited chemostat cultures. *J. Gen. Microbiol.* (1990) doi:10.1099/00221287-136-3-405.

60. Van Hoek, P., Van Dijken, J. P. & Pronk, J. T. Effect of specific growth rate on fermentative capacity of baker's yeast. *Appl. Environ. Microbiol.* **64**, 4226–33 (1998).

61. Nissen, T. L., Schulze, U., Nielsen, J. & Villadsen, J. Flux distributions in anaerobic, glucose-limited continuous cultures of Saccharomyces cerevisiae. *Microbiology* (1997) doi:10.1099/00221287-143-1-203.

62. Snitkin, E. S. *et al.* Model-driven analysis of experimentally determined growth phenotypes for 465 yeast gene deletion mutants under 16 different conditions. *Genome Biol.* **9**, R140 (2008).

63. Bakker, B. M. *et al.* Stoichiometry and compartmentation of NADH metabolism in Saccharomyces cerevisiae. in *FEMS Microbiology Reviews* (2001). doi:10.1016/S0168-6445(00)00039-5.

64. Garlid, K. D., Beavis, A. D. & Ratkje, S. K. On the nature of ion leaks in energy-transducing membranes. *BBA - Bioenergetics* (1989) doi:10.1016/S0005-2728(89)80219-1.

65. Fitton, V., Rigoulet, M., Ouhabi, R. & Guérin, B. Mechanistic Stoichiometry of Yeast Mitochondrial Oxidative Phosphorylation. *Biochemistry* (1994) doi:10.1021/bi00198a039.

66. Rigoulet, M., Mourier, A., Galinier, A., Casteilla, L. & Devin, A. Electron competition process in respiratory chain: Regulatory mechanisms and physiological functions. *Biochim. Biophys. Acta - Bioenerg.* (2010) doi:10.1016/j.bbabio.2010.01.030.

67. Ye, L., Berden, J. A., Van Dam, K. & Kruckeberg, A. L. Expression and activity of the Hxt7 high-affinity hexose transporter of Saccharomyces cerevisiae. *Yeast* **18**, 1257–1267 (2001).

68. Kruckeberg, A. L., Ye, L., Berden, J. A. & van Dam, K. Functional expression, quantification and cellular localization of the Hxt2 hexose transporter of Saccharomyces cerevisiae tagged with the green fluorescent protein. *Biochem. J.* **339 (Pt 2**, 299–307 (1999).

69. Shokrollahzadeh, S., Bonakdarpour, B., Vahabzadeh, F. & Sanati, M. Growth kinetics and Pho84 phosphate transporter activity of Saccharomyces cerevisiae under phosphate-limited conditions. *J. Ind. Microbiol. Biotechnol.* **34**, 17–25 (2007).

70. Chemical, C. *et al.* Global analysis of protein expression in yeast. **108**, 737–741 (2003).

71. Wacker, T., Garcia-Celma, J. J., Lewe, P. & Andrade, S. L. Direct observation of electrogenic NH4(+) transport in ammonium transport (Amt) proteins. *Proc Natl Acad Sci U S A* **111**, 9995–10000 (2014).

72. de Groot, D. H., van Boxtel, C., Planqué, R., Bruggeman, F. J. & Teusink, B. The number of active metabolic pathways is bounded by the number of cellular constraints at maximal metabolic rates. *PLoS Comput. Biol.* **15**, e1006858 (2019).

73. Wortel, M. T., Peters, H., Hulshof, J., Teusink, B. & Bruggeman, F. J. Metabolic states with maximal specific rate carry flux through an elementary flux mode. *FEBS J.* **281**, 1547–1555 (2014).

74. de Groot, D. H., Hulshof, J., Teusink, B., Bruggeman, F. J. & Planqué, R. Elementary Growth Modes provide a molecular description of cellular self-fabrication. *PLoS Comput. Biol.* (2020) doi:10.1371/journal.pcbi.1007559.

75. Basan, M. *et al.* Overflow metabolism in Escherichia coli results from efficient proteome allocation. *Nature* **528**, 99–104 (2015).

76. Schuster, S., Dandekar, T. & Fell, D. A. Detection of elementary flux modes in biochemical networks: A promising tool for pathway analysis and metabolic engineering. *Trends Biotechnol.* **17**, 53–60 (1999).

77. Terzer, M. & Stelling, J. Large-scale computation of elementary flux modes with bit pattern trees. *Bioinformatics* (2008) doi:10.1093/bioinformatics/btn401.

78. Bruggeman, F. J., Planqué, R., Molenaar, D. & Teusink, B. Searching for principles of microbial physiology. *FEMS Microbiology Reviews* (2020) doi:10.1093/femsre/fuaa034.

79. Kafri, M., Metzl-Raz, E., Jona, G. & Barkai, N. The Cost of Protein Production. *Cell Rep.* **14**, 22–31 (2016).

80. Waldron, C., Jund, R. & Lacroute, F. Evidence for a high proportion of inactive ribosomes in slow growing yeast cells. *Biochem. J.* (1977) doi:10.1042/bj1680409.

81. Spahn, C. M. T. *et al.* Structure of the 80S ribosome from Saccharomyces cerevisiae - tRNA-ribosome and subunit-subunit interactions. *Cell* (2001) doi:10.1016/S0092-8674(01)00539-6.

82. Panse, V. G. & Johnson, A. W. Maturation of eukaryotic ribosomes: acquisition of functionality. *Trends in Biochemical Sciences* (2010) doi:10.1016/j.tibs.2010.01.001.
