# Supplementary material for: Whole-cell modeling in yeast predicts compartment-specific proteome constraints that drive metabolic strategies
Source: Nat Commun. 2022 Feb 10;13:801. doi: 10.1038/s41467-022-28467-6 (PMC8831649; doi:10.1038/s41467-022-28467-6)
Supplement: Supplementary file 3 — Description of Additional Supplementary Files [file 41467_2022_28467_MOESM3_ESM.pdf]

Description of Additional Supplementary Files

Supplementary Data 1.

Description: Flux and yield data of yeast cultures, grown in glucose-limited chemostats, excess sugar batches and in glucose-excess batches treated with cycloheximide.

Supplementary Data 2.

Description: Label-free quantitative proteomics data for the cultures, grown in glucose-limited chemostats.

Supplementary Data 3.

Description: Label-free quantitative proteomics data for the cultures, grown in excess sugar batches.

Supplementary Data 4.

Description: Label-free quantitative proteomics data for the cultures, grown in excess glucose batches, treated with cycloheximide.

Supplementary Data 5.

Description: Protein-pathway and protein-compartment mappings, used in the study.

Supplementary Data 6.

Description: Lists of genes, activated by PKA-, TOR-, or cooperative PKA and TOR signalling.
