# Supplementary material for: Whole-cell modeling in yeast predicts compartment-specific proteome constraints that drive metabolic strategies
Source: Nat Commun. 2022 Feb 10;13:801. doi: 10.1038/s41467-022-28467-6 (PMC8831649; doi:10.1038/s41467-022-28467-6)
Supplement: Supplementary file 10 — Reporting Summary [file 41467_2022_28467_MOESM10_ESM.pdf]

nature portfolio

Corresponding author(s): Bas Teusink

Last updated by author(s): Dec 1, 2021

Reporting Summary

Nature Portfolio wishes to improve the reproducibility of the work that we publish. This form provides structure for consistency and transparency in reporting. For further information on Nature Portfolio policies, see our Editorial Policies and the Editorial Policy Checklist.

Statistics

For all statistical analyses, confirm that the following items are present in the figure legend, table legend, main text, or Methods section.

n/a	Confirmed	
☐	☒	The exact sample size (*n*) for each experimental group/condition, given as a discrete number and unit of measurement
☐	☒	A statement on whether measurements were taken from distinct samples or whether the same sample was measured repeatedly
☒	☐	The statistical test(s) used AND whether they are one- or two-sided<br>*Only common tests should be described solely by name; describe more complex techniques in the Methods section.*
☒	☐	A description of all covariates tested
☒	☐	A description of any assumptions or corrections, such as tests of normality and adjustment for multiple comparisons
☒	☐	A full description of the statistical parameters including central tendency (e.g. means) or other basic estimates (e.g. regression coefficient) AND variation (e.g. standard deviation) or associated estimates of uncertainty (e.g. confidence intervals)
☒	☐	For null hypothesis testing, the test statistic (e.g. *F*, *t*, *r*) with confidence intervals, effect sizes, degrees of freedom and *P* value noted<br>*Give P values as exact values whenever suitable.*
☒	☐	For Bayesian analysis, information on the choice of priors and Markov chain Monte Carlo settings
☒	☐	For hierarchical and complex designs, identification of the appropriate level for tests and full reporting of outcomes
☒	☐	Estimates of effect sizes (e.g. Cohen's *d*, Pearson's *r*), indicating how they were calculated

Our web collection on statistics for biologists contains articles on many of the points above.

Software and code

Policy information about availability of computer code

Data collection	Model development and handling: linear program solvers IBM ILOG CPLEX 12.10 and SoPlex 4.0.2; MATLAB version of the model: MATLAB 2016a; Python version of the model: Python 2.7 and pip packages cbmpy, pandas, numpy.<br>Mass spectrometry: QExactive HF Tune 2.5.0.2042, XCalibur 3.1<br>The model implementation in Python, together with the code and data used to make figures of this manuscript (based on the Python implementation) are provided in ZENODO repository (doi 10.5281/ZENODO.5732995). Legacy model implementation in MATLAB is available on GitHub, SysBioChalmers/Yeast-ME-GEM.
Data analysis	General data handling: Microsoft Excel 2016.<br>Analysis of model output and plotting: Python 2.7 and packages pandas, numpy, matplotlib.<br>Analysis of mass spectrometry data: MaxQuant 1.6.0.16; R 3.3.3 with the MSstats 3.5.6 package.

For manuscripts utilizing custom algorithms or software that are central to the research but not yet described in published literature, software must be made available to editors and reviewers. We strongly encourage code deposition in a community repository (e.g. GitHub). See the Nature Portfolio guidelines for submitting code & software for further information.

Data

Policy information about availability of data

All manuscripts must include a data availability statement. This statement should provide the following information, where applicable:

- Accession codes, unique identifiers, or web links for publicly available datasets
- A description of any restrictions on data availability
- For clinical datasets or third party data, please ensure that the statement adheres to our policy

Physiological measurements (specific consumption and secretion rates and yields) are provided in the Supplementary Dataset 1. Processed label-free quantitative proteomics data of the chemostat and bioreaction cultivations are provided in Supplementary Datasets 2 to 4. Raw mass spectrometry data are available at the PRIDE database with identifier PXD030003. Source data for reproducing Figures 1-5 are provided with this paper.

Field-specific reporting

Please select the one below that is the best fit for your research. If you are not sure, read the appropriate sections before making your selection.

- [x] Life sciences
- [ ] Behavioural & social sciences
- [ ] Ecological, evolutionary & environmental sciences

For a reference copy of the document with all sections, see nature.com/documents/nr-reporting-summary-flat.pdf

Life sciences study design

All studies must disclose on these points even when the disclosure is negative.

Sample size	No sample-size calculation was performed. Chemostat and bioreactor cultivations were perfomed in biological duplicates. Parameters, such as temperature and pH, were continuously measured during cultivations and appropriate responses were handled by the computer-based controller, thus the variability between cultivations is expected to be low.
Data exclusions	The experiments of the bioreactor with the highest concentration of translational inhibitor (lowest specific growth rate) (experiments presented in Fig. 5a of the main text) were excluded from further analyses based on inspection of growth curves.
Replication	Cell cultivations were performed in well-controlled environments (chemostats and biorectors, computer-controlled), all in biological duplicates. Physiological parameters, such as dynamics of optical density over time, were assessed between duplicates before proceeding with analytical measurements and MS-based proteomics.
Randomization	Randomization not relevant for cell cultivations; the run order of MS-based proteomics samples was randomized.
Blinding	Blinding not relevant for cell cultivations, and the sample running order for MS-based proteomics was randomized (see above).

Reporting for specific materials, systems and methods

We require information from authors about some types of materials, experimental systems and methods used in many studies. Here, indicate whether each material, system or method listed is relevant to your study. If you are not sure if a list item applies to your research, read the appropriate section before selecting a response.

Materials & experimental systems

n/a	Involved in the study
x	Antibodies
x	Eukaryotic cell lines
x	Palaeontology and archaeology
x	Animals and other organisms
x	Human research participants
x	Clinical data
x	Dual use research of concern

Methods

n/a	Involved in the study
x	ChIP-seq
x	Flow cytometry
x	MRI-based neuroimaging
